# Stem cells in clinical practice: applications and warnings

**DOI:** 10.1186/1756-9966-30-9

**Published:** 2011-01-17

**Authors:** Daniele Lodi, Tommaso Iannitti, Beniamino Palmieri

**Affiliations:** 1 Department of Nephrology, Dialysis and Transplantation, University of Modena and Reggio Emilia Medical School, Modena, Italy; 2 Department of Biological and Biomedical Sciences, Glasgow Caledonian University, Glasgow, UK; 3 Department of General Surgery and Surgical Specialties, University of Modena and Reggio Emilia Medical School, Surgical Clinic, Modena, Italy

## Abstract

Stem cells are a relevant source of information about cellular differentiation, molecular processes and tissue homeostasis, but also one of the most putative biological tools to treat degenerative diseases. This review focuses on human stem cells clinical and experimental applications. Our aim is to take a correct view of the available stem cell subtypes and their rational use in the medical area, with a specific focus on their therapeutic benefits and side effects. We have reviewed the main clinical trials dividing them basing on their clinical applications, and taking into account the ethical issue associated with the stem cell therapy.

**Methods:**

We have searched Pubmed/Medline for clinical trials, involving the use of human stem cells, using the key words "stem cells" combined with the key words "transplantation", "pathology", "guidelines", "properties" and "risks". All the relevant clinical trials have been included. The results have been divided into different categories, basing on the way stem cells have been employed in different pathological conditions.

## Introduction

The word "stemness" defines a series of properties which distinguish a heterogeneous variety of cell population. However, in the absence of a current consensus on a gold standard protocol to isolate and identify SCs, the definition of "stemness" is in a continuous evolution [1-3].

Biologically, stem cells (SCs) are characterized by self-renewability [4], that is the ability not only to divide themselves rapidly and continuously, but also to create new SCs and progenitors more differentiated than the mother cells. The asymmetric mitosis is the process which permits to obtain two intrinsically different daughter cells. A cell polarizes itself, so that cell-fate determinant molecules are specifically localized on one side. After that, the mitotic spindle aligns itself perpendicularly to the cell axis polarity. At the end of the process two different cells are obtained [5-7].

SCs show high plasticity, i.e. the complex ability to cross lineage barriers and adopt the expression profile and functional phenotypes of the cells that are typical of other tissues. The plasticity can be explained by transdifferentiation (direct or indirect) and fusion. Transdifferentiation is the acquisition of the identity of a different phenotype through the expression of the gene pattern of other tissue (direct) or through the achievement of a more primitive state and the successive differentiation to another cell type (indirect or de-differentiation). By fusion with a cell of another tissue, a cell can express a gene and acquire a phenotypic element of another parenchyma [3].

SCs morphology is usually simpler than that one of the committed cells of the same lineage. It has often got a circular shape depending on its tissue lineage and a low ratio cytoplasm/nucleus dimension, i.e. a sign of synthetic activity. Several specifics markers of general or lineage "stemness" have been described but some, such as alkaline phosphatase, are common to many cell types [1,8-11].

From the physiological point of view, adult stem cells (ASCs) maintain the tissue homeostasis as they are already partially committed. ASCs usually differentiate in a restricted range of progenitors and terminal cells to replace local parenchyma (there is evidence that transdifferentiation is involved in injury repair in other districts [12], damaged cells or sustaining cellular turn over [13]). SCs derived from early human embryos (Embryonic stem cells (ESCs)), instead, are pluripotent and can generate all committed cell types [14,15]. Fetal stem cells (FSCs) derive from the placenta, membranes, amniotic fluid or fetal tissues. FSCs are higher in number, expansion potential and differentiation abilities if compared with SCs from adult tissues [16]. Naturally, the migration, differentiation and growth are mediated by the tissue, degree of injury and SCs involved. Damaged tissue releases factors that induce SCs homing. The tissue, intended as stromal cells, extracellular matrix, circulating growth and differentiating factors, determines a gene activation and a functional reaction on SCs, such as moving in a specific district, differentiating in a particular cell type or resting in specific niches. These factors can alter the gene expression pattern in SCs when they reside in a new tissue [17].

Scientific research has been working to understand and to indentify the molecular processes and cellular cross-talking that involve SCs. Only with a deep knowledge of the pathophysiological mechanism involving SCs, we might be able to reproduce them in a laboratory and apply the results obtained in the treatment of degenerative pathologies, i.e. neurological disorder such as Parkinson's disease (PD), Alzheimer's disease (AD), Huntington's disease, multiple sclerosis [18], musculoskeletal disorder [19], diabetes [20], eye disorder [21], autoimmune diseases [22], liver cirrhosis [23], lung disease [24] and cancer [25].

In spite of the initial enthusiasm for their potential therapeutic application, SCs are associated with several burdens that can be observed in clinical practice. Firstly, self-renewal and plasticity are properties which also characterize cancer cells and the hypothesis to lose control on transplanted SCs, preparing a fertile ground for tumor development, is a dangerous and unacceptable side effect [26,27]. Secondly, in case of allogenic SCs graft, several cases of immunorejection or graft versus host disease [28] are reported, with a necessary immunosuppressive treatment to avoid immune response against the transplant and the consequent risk of infections. Finally, to succeed in ESCs cultures, it is necessary to manipulate and to reproduce embryos for scientific use, but the Catholic World identifies this stage of the human development with birth and attributes embryos the same rights [29].

## Stem Cells Types

SCs are commonly defined as cells capable of self-renewal through replication and differentiating into specific lineages. Depending on "differentiating power", SCs are divided into several groups. The cells, deriving from an early progeny of the zygote up to the eight cell stage of the morula, are defined as "totipotent", due to their ability to form an entire organism [30]. The "pluripotent" cells, such as ESCs, can generate the tissues of all embryonic germ layers, i.e. endoderm, mesoderm, and ectoderm, while "multipotent" cells, such as ASCs, are capable of yielding a more restricted subset of cell lineages. Another type of SCs classification is based on the developmental stage from which they are obtained, i.e. embryonic origin (ESCs) or postnatal derivation (ASCs) [3].

### Embryo-derived stem cells

A zygote is the initial cell originating when a new organism is produced by means of sexual reproduction. Zygotes are usually produced by a fertilization event between two haploid cells, i.e. an ovum from a female and a sperm cell from a male, which combine to form the single diploid cell [31].

The blastocyst is the preimplantation stage in embryos aged one week approximately. The blastocyst is a cave structure compound made by the trophectoderm, an outer layer of cells filling cavity fluid and an inner cell mass (ICM), i.e. a cluster of cells on the interior layer [32-35].

Embryonic cells (EC, epiblast) are contained in the ICM and generate the organism, whereas the surrounding trophoblast cells contribute to the placental chorion. Traditionally, ECs are capable of a self-renewal and differentiation into cells of all tissue lineages[15], but not into embryonic annexes as such zygote. ECs can be cultured and ESCs can be maintained for a long time (1-2 years with cell division every 36-48 hours) in an undifferentiated phenotype [10,33,36] and which unchanged properties. ECs can be isolated by physical micro dissection or by complement-mediated immune dissection. ECs are preserved through fast freeze or vitrification techniques to avoid an early natural differentiation [37-39]. Culturing ESCs requires a special care, in fact, under SCs, a feeder layer of primary murine fibroblast is seeded in a permanent replication block that sustains continuously undifferentiated ESCs [14]. ESCs are maintained for a long time in culture to obtain a large pool of undifferentiated SCs for therapeutic and research applications. In contrast, somatic cells and mesenchimal stem cells (MSCs) have finite replicative lifespan after which they can no longer divide and are said to have reached a proliferative senescence [40]. The replicative lifespan of cells depends on the cell type, donor's species, and donor's age, but it is directly related to telomerase activity [41-44]. Telomerase is an enzyme which adds specific short sequences to chromosomes ends, aiming at preserving chromosome length and supporting the ongoing cell division [42]. Telomerase activity is decreased by committing and, as a result, it is characteristically high in ESCs, intermediate in haematopoietic stem cells (HSCs), and variable, or even absent, in somatic cells [3,42].

### Fetal stem cells

FSCs are multipotent cells with the same functional properties of ASCs, but they locate in the fetal tissue and embryonic annexes. Indeed, further analyses are necessary to investigate whether ASCs are the same present in the tissue. FSCs have been subdivided into haemopoietic ones, located in blood, liver, bone marrow (BM), mesenchymal ones located in blood, liver, BM, lung, kidney and pancreas, endothelial ones found in BM and placenta, epithelial ones located in liver and pancreas and neural ones located in brain and spinal cord [45]. Obviously, the only source of FSCs, relatively feasible and safe for fetus, is fetal blood [46]. Nowadays a routine procedure for fetal diagnosis and therapy, which are the most diffuse techniques to harvest FSCs, is ultrasound guided accession to fetal circulation [45].

### Adult stem cells

ASCs are partially committed SCs localized in specific stromal niches. ASCs can be obtained from the mesodermal tissues such as BM [1,47], muscle [48], adipose tissue [49], synovium [50] and periosteum [51]. SCs have been also isolated from the tissues of endodermal lineages such as intestine [52] and from the ectodermal tissues including skin [53], deciduous teeth [54] and nerve tissue [8,9,55,56]. ASCs originate during ontogenesis and remain in a marginal area in a quiescent state as the local stimuli induce their cycle recruitment and migration. In fact, niche microenvironment, with physical contact and chemical dialogue among SCs, stromal cells and matrix, induce ASCs differentiation and self-renewal [57,58].

Probably, for documented plasticity and easy extraction, several ASCs types, such as HSCs, adipose tissue-derived stromal cells (ADSCs) and derived MSCs, have had and have a historical importance. HSCs are well characterized cells of mesodermal origin deriving prevalently from BM, in particular near endosteal bone surface and sinusoidal endothelium and from peripheral blood. Traditionally HSCs generate all mature blood cell types of the hematolymphatic system including neutrophils, monocytes/macrophages, basophils, eosinophils, erythrocytes, platelets, mast cells, dendritic cells, and B and T lymphocytes. More recently, HSCs have shown to display remarkable plasticity and can apparently differentiate into several non-hemolymphatic tissue lineages [3]. The identification and isolation of HSCs is possible with immune capture of CD34, a surface protein that distinguishes SCs from other hematopoietic cells [59]. HSCs are at the base of BM transplant procedures, i.e. myeloablation or adiuvant therapy where HSCs are infused in the recipient [60].

MSCs originally derive from BM, [1,8,47] but they have been isolated from other tissues, such as adipose tissue, periosteum, synovial membrane, synovial fluid (SF), muscle, dermis, deciduous teeth, pericytes, trabecular bone, infrapatellar fat pad, and articular cartilage [1,19,47,61-68]. They are generally restricted to forming only mesodermal-specific cell types such as adipocytes, osteoblasts, myocytes and chondrocytes, but several MSCs are able to differentiate in cells of the three embryonic germ layers [69]. Several of these studies report the differentiation of MSCs into various tissue lineages in vitro and the repair or "engraftment" of the damaged organs in vivo, such as bone tissue repair and immune system reconstruction, but they are even able to differentiate in endothelial cells and contribute to revascularization of the ischemic tissue [3,70,71]. In particular, recent studies show that cultured MSCs secrete various bioactive molecules which have got anti-apoptotic, immunomodulatory, angiogenic, anti-scarring and chemo-attractant properties, providing a basis for their use as tools to create local regenerative environments in vivo [72].

### Umbilical cord stem cells

In the umbilical cord, we can find two types of SC sources, i.e. the umbilical cord epithelium (UCE), derived from the amniotic membrane epithelium and the umbilical cord blood (UCB) [73]. Although its general architecture significantly differs from the mammalian epidermis, UCE expresses a cytokeratin pattern similar to human epidermis [74,75]. UCE is able to form a stratified epithelium when seeded on fibroblast populated collagen gels [76,77]. It has been demonstrated that UCE is an important source of the human primary keratinocytes and it is able to recreate the epidermis for dermatological application [78]. In UCB we can find two different types of SCs, i.e. hematopoietic (UC-HS) and mesenchymal (UC-MS). Although UCB SCs are biologically analogous to their adult counterpart, it has been pointed out that UCB cells are characterized by a higher immunological tolerance than their adult counterpart [79]. Indeed UC-MS can produce cytokines which facilitate grafting in the donor, in vitro SC survival and it is more efficient than BM MSC graft [80].

## Risks And Obstacles To Stem Cells Application In Clinical Practice

### Risks

SC graft induces therapeutic and side effects. A specific evaluation of the side effects is needed to decide if a cure can be adopted in medical practice. Indeed, scientific research has to outline the severity of undesired effects, their frequency in treated subjects and the possibility to avoid, reduce or abate them. The major limitations to the success of HSC transplantation (HSCT) are respiratory complications and graft versus host disease. Lung dysfunction occurs in up to 50% of the subjects after HSCT, and pulmonary complications are among the most common causes of morbidity and mortality after this procedure.

Obliterative bronchiolitis (OB) is a multifactorial process involving both alloimmunologic and nonalloimmunologic reactions as the heterogeneous histopathologic findings and clinical course suggest. Since the occurrence of OB has been closely associated with GVHD, it has been hypothesized that OB is mediated, partially, by alloimmunologic injury to host bronchiolar epithelial cells [81-83]. Usually, OB develops as a late complication, i.e. after the first 100 days, of HSCT. The OB onset is usually 6-12 months post-transplant, with the clinical seriousness ranging from asymptomatic severity to a fulminant and fatal one. The pathogenesis of the disease is believed to primarily involve the interplay among immune effectors cells that have been recruited from the lung and cells resident in the pulmonary vascular endothelium and interstitium. This complex process results in the loss of type I pulmonary epithelial cells, a proliferation of type II cells, the recruitment and proliferation of endothelial cells and the deposition of the extracellular matrix. In response to the pattern of injury, cytokines are released from immune effectors cells and lung cells, i.e. macrophages, alveolar epithelial, and vascular endothelial cells, and they can stimulate the fibroblast proliferation and increase the synthesis of collagen and extracellular matrix proteins. The result is the large deposition of collagen and granulation tissue in and around the bronchial structures, with the partial or complete small airway obliteration. Clinical data suggest that nonalloimmunologic inflammatory conditions, such as viral infections, recurrent aspiration, and conditioning chemoradiotherapy may also play a role in the pathogenesis of OB after HSC transplantation [84,85]. Bronchiolitis obliterans organizing pneumonia (BOOP) is a disorder involving bronchioles, alveolar ducts, and alveoli, whose lumen becomes filled with buds of granulation tissue, consisting of fibroblasts and an associated matrix of loose connective tissue. It derives from the proliferative type, and it generally includes mild inflammation of the bronchiolar walls. In contrast to BO, there is no prominent bronchiolar wall fibrosis or bronchiolar distortion [86]. The involvement of an alloimmunologic reaction can be considered, although the pathogenesis of BOOP following HSCT is poorly understood. In animal studies, BOOP develops after a reovirus infection. A significant role for T cells and Th1-derived cytokines, including interferon-α, is implicated in the development of disease [87]. Indeed, T-cell depletion prevents from BO and BOOP after allogeneic hematopoietic SC transplantation with related donors [88]. A reported case, following syngeneic BM transplantation, suggests that BOOP is not always the result of an allogeneic immune response [89]. In other non-HSCT settings, BOOP has been seen in association with infection, drugs, radiation therapy, and a number of connective tissue disorders [90]. It has also been shown that the 2-year cumulative incidence of late-onset non-infectious pulmonary complications (LONIPC, including BO and BOOP) has been 10% in 438 patients undergoing HSCT. Moreover, the survival rate at 5 years has been significantly worse in affected subjects than in unaffected ones [91].

Graft versus host disease (GVHD) is a frequent and lethal complication of HSCT that limits the use of this important therapy. On the basis of pathophysiology and appearance, GVHD is classified in acute and chronic one [92]. Acute GVHD occurs prior to day 100 after transplant and it consists in an enhanced inflammatory/immune response, mediated by the competent donor's lymphocytes, infused into the recipient, where they react against an environment perceived as a foreign one. The process is amplified through the tissue release of molecules which stimulate the donor's lymphocytes. This apparently contradictory phenomenon is simply a physiological reaction of the damaged tissue to the disease which has led to the transplant therapy [93]. Acute GVHD presents clinical manifestations in the skin, i.e. maculopapular rash, which can spread throughout the body, dyskeratosis (in severe cases the skin may blister and ulcerate) [94], in the gastrointestinal tract, i.e. diarrhea, emesis, anorexia, abdominal pain, mucosal ulceration with bleeding, luminal dilatation [95], and in the liver, i.e. same liver dysfunction of veno-occlusive disease, drug toxicity, viral infection, sepsis, or iron overload [96]. Chronic GVHD is the major cause of late non-relapse death following HCT [97]. However, chronic GVHD pathophysiology is not completely understood. Probably, thymus atrophy or dysfunction, which can develop after pharmacological preparation of transplant, play a major role in chronic GVHD manifestation. This fact leads to a peripheral tolerance decrease and to an increase in the number of autoreactive T lymphocytes. Autoreactive T lymphocytes lead to an interferon gamma mediated increase in the collagen deposition and fibrosis, a characteristic feature of chronic GVHD [97,98]. The manifestations of chronic GVHD are protean and often of an autoimmune nature. Many districts are involved, i.e. skin with dyspigmentation, alopecia, poikiloderma, lichen planus-like eruptions or sclerotic features, nails with nail dystrophy or loss, the mouth with xerostomia, ulcers, lichen-type features, restrictions of mouth opening from sclerosis, eyes with dry eyes, sicca syndrome, cicatricial conjunctivitis, muscles, fascia and joints with fasciitis, myositis, or joint stiffness from contractures, the female genitalia with vaginal sclerosis, ulcerations, the gastrointestinal tract with anorexia, weight loss, esophageal web or structures, liver with jaundice, transaminitis, lungs with restrictive or obstructive defects on pulmonary function tests, bronchiolitis obliterans, pleural effusions, kidneys with nephrotic syndrome (rare), heart with pericarditis and bone marrow (thrombocytopenia, anemia, neutropenia) [92,99,100].

Hepatic veno-occlusive disease (VOD) is another recurrent complication after SC transplantation. VOD is a condition in which some of the small hepatic veins are blocked, in this case, by cells. It is a complication of high-dose chemotherapy given before a BM transplant and it is marked by weight gain, due to fluid retention, increased liver size, and raised levels of bilirubin in the blood [101,102]. VOD is more frequent in children undergoing SC transplantation [103].Two hundred and forty four HSCTs have been evaluated and it has been found that VOD had appeared in 11% of them. It has been identified that risk factors for VOD are age <6.7 years, type of VOD prophylaxis, and busulphan-containing conditioning regimens [104]. Interesting results have been obtained in VOD treatment by oral defibrotide [105] and combination of intravenous heparin, oral glutamine and ursodiol [106].

### Obstacles and possible solutions

The compatibility between the recipient and the graft is the main problem that must be faced off when a medical group decides to transplant organs, tissues or cells successfully. In SCT, the immunorejection also represents an important obstacle. If autogenous cells are available, immunorejection can be bypassed. In fact, common clinical practice is to harvest autogenous MCSs, expand them in culture, avoiding microorganism contamination, and store the obtained cell population before implantation [9].

Interestingly, allogenic MCSs transplant, obviously applied in emergency situations, such as spinal cord injury or myocardial infarction, demonstrates high success rates. A tolerance of allogenic MCSs seems to be induced by the same grafted cells. Indeed, MCSs inhibit T cell proliferation and maturation through direct cell-cell effects and by secretion of soluble factors [107,108].

Allogenous EC transplantation is not immunotolerated as MSCs graft. Therefore, avoiding the EC immunorejection, several strategies are being developed. Somatic cell nuclear transfer (SCNT) is currently the most promising of them. SCNT consists in the enucleation of the donor's oocytes and the renucleation of them with nuclei taken from the patient's somatic cells. The created cells are tolerated because they express major histocompatability complex (MHC) of the recipient. The disadvantages of SCNT include the creation and destruction of embryos and the current inability to apply the technology in autoimmune diseases [109]. In order to avoid autoimmune rejection, some elaborate methods, such as gene therapy, are under investigation [3,110].

ESCs are characterized by genetic instability and imprinting genes dysregulation [111]. Indeed, their transplantation in rodents is associated to higher risk of malignant transformations, such as teratomas or teratocarcinomas [112-114], although the tumorigenic potential of ESC seems to be greatly reduced when the cells are predifferentiated in vitro before implantation [115]. The graft of ESCs must be preceded by an accurate functional characterization to distinguish partially transformed and potentially oncogenic clones and normal cells [116].

### Medical tourism

In developing countries some doctors are treating patients with ASC without waiting for clinical trials to validate the safety of using them for health problems [117].

In treatments, involving the use of ASC, the cells are injected into the blood, lumbar region, or damaged tissue. The only treatments using ASC that are proven by clinical trials, are concerned with blood disorders, bone marrow transplantation and rare immune deficiencies. Several cases of patients, who developed serious side effects following SC transplantation, such as brain tumors, after injections of fetal neural SC, or meningitis have been reported[118].

A Google search, using the key words ''stem cell therapy'' or ''treatment'', has outlined the range of treatments being offered directly to consumers. Websites generally describe therapies as safe, effective, and ready for routine use in a wide variety of conditions. In contrast, the published clinical evidence has been unable to support the use of these therapies for the routine disease treatment. Patients must receive sufficient and appropriate information and fully understand the risks. Clinics must also contribute to public expectations without exceeding what the field can reasonably achieve. However, this interpretation is subject to the following limitations: information, available from websites, could not be indicative of the information actually shared with patients during their clinical encounters; the aggregate data, collected from a heterogeneous group of clinics, could not be used to evaluate the claims of any particular clinic; and finally, the accuracy of websites' claims has not been tested directly by analyzing actual outcome data. Instead, there is a lack of high quality evidence supporting SC clinics' claims. Even supposing that clinics have indeed observed successful recovery from chronic disease post-treatment, a lack of good evidence precludes a valid or precise inference that the observed improvement is attributable to the interventions. If, in fact, the interventions had not been effective, then the patients would have been subjected to inappropriate risks and exaggerated financial burden [119,120].

## Possible Clinical Uses

### Autoimmune disease

#### Rheumatoid arthritis and juvenile idiopathic arthritis

Rheumatoid arthritis **(**RA) is the progressive and irreversible erosion of the cartilage tissue of joint with the consequent loss of mobility, pain and reduction in the quality of life. Probably, RA and juvenile idiopathic arthritis (JIA) are caused by failure of tolerance and immune response against joint tissue antigens and aptens with abundant release of inflammatory cytokines and autoantibody [121,122]. Standard therapy encloses nonsteroidal medications with slow addition of traditional disease-modifying anti-rheumatic drugs (DMARDs) or intra-articular corticosteroid injections, but the remission rate is only about 15% [123].

Several clinical trials have been conducted to treat RA and JIA with autologous HSCs transplantation (AHSCT).

A significant response has been obtained in most subjects in a study involving 76 patients with severe RA which were resistant to conventional therapies and submitted to AHSCT. Although the disease has not been cured, recurrent or persistent disease activity has been controlled, in some cases, with common antirheumatic drugs [124]. A trial, involving 33 patients with severe, refractory RA, randomly submitted to either AHSCT or selected CD34+ infusion, has not shown any advantage with antigen selection, but it has confirmed immunomodulatory action of HSC in joint microenvironment [125]. A successfully HSCT protocol has been proposed to treat severe JIA, harvest BM, select positive SCs, deplete T cells, re-infuse the cells and administer antiviral drugs and immunoglobuline until the immune system returns to full competence to avoid frequent infection [126].

#### Systemic lupus erythematosus

Systemic lupus erythematosus (SLE) is a multi-system, inflammatory, autoimmune disease, caused by BM microenvironment dysfunction and consequently a marked reduction of number and proliferative capability of HSCs with a hyperproduction of immunocomplex. Cells CD34+ undergo an elevated apoptosis rate. SLE includes nephritis, serositis, pneumonitis, cerebritis, vasculitis, anti-phospholipid antibody syndrome with venous and vascular thrombi, arthalgias, myalgias, cutaneous symptoms [127]. Usually SLE is aspecifically treated with non-steroidal anti-inflammatory drugs, antimalarials, corticosteroids and cytotoxic agents. However, every drug involves severe side effects and frequent relapses [128].

AHSCT has reduced the number of apoptotic CD34+ cells pre-treatment [22]. In the last decade, contrasting results have been reported in literature. AHSCT has been performed on 15 patients with severe SLE with a general positive outcome. Only two subjects have had a recurrence of symptoms [129]. However, it has been reported a lower disease free rate and high mortality [130]. Further trials are required, but it seems probable that HSCT can be used not with a curative intent, but to mitigate the disease impact towards a more drug sensitive type. However, it should be reserved only for those patients with persistence of organ-threatening SLE, despite the standard aggressive therapy [131].

#### Multiple sclerosis

Multiple Sclerosis (MS) is a life-threatening, physically and psychologically debilitating autoimmune disease

(AD), mediated by T cells triggered against structural components of myelin and consequent degenerative loss of axon in the central nervous system (CNS). In fact, the nerve atrophy progressively reduces the electrical signalling neurons muscles and related mobility. The inflammatory reaction is an important component of MS physiopathology and the conventional treatments aims at reducing it in order to cure or postpone course disease [132,133]. Two types of MS can be identified: primary progressive MS (PPMS), generally resistant to treatment and without amelioration, and secondary progressive MS (SPMS) with episodic relapse and improvement [134].

As gold standard therapy efficiently delays MS progression for many years, AHSCT have been performed on patients who do not respond to conventional therapies, and consequently the results have not been encouraging and, in several cases, they have taken a turn for the worse [135]. Furthermore, graft exposes patients to infection risks, localized toxicity or autoimmune diseases [136,137]. However, it has been reported a reduction of CNS inflammation with a stabilization of the disease in patients aged less than 40 years [136]. A plastic conversion of HSC-derived cells, to replace damage neurons, has been hypothesized [138].

#### Systemic sclerosis

Systemic sclerosis (SSc) is a multisystem, rare disorder characterized by cutaneous and visceral (pulmonary, cardiac, gastrointestinal and renal) fibrosis as a consequence of T cell activation, autoantibody production, cytokine secretion and excessive collagen deposition. Patients with the diffuse variant, who have extensive skin and early visceral involvement, have a poor outcome with a 5-year mortality which is estimated at 40-50% in 5 years [139]. The therapy for the SSc is far from being perfect. At present, the best results are obtained with the combination of cyclophosphamide (CY) and angiotensin [140].

It has been demonstrated that AHSCT improves the skin flexibility and stabilizes the pulmonary involvement [141-146].

Farge et al. have compared two studies with conflicting results. The first describes a long time remission rate of 80% (partial or complete) on 57 patients, and the majority of the subjects have presented a general improvement of pre-AHSCT clinical condition. The second study, instead, shows a higher reactivation rate (50%). Interestingly, AHSCT can extend the short life expectancy of patients with severe SS [147].

Ultimately, priming regimens, i.e. a disease progression and transplant procedure, that is transplanted-related complication, have been associated to high mortality rates (27%) [143].

### Crohn's disease

It is an incompletely known autoimmune disease characterized by the gastrointestinal loss of immune tolerance caused by overactive T-helper 1 response. The environmental agents and genetic factors are also involved. Sometimes the disease can be controlled by immunosuppressive drugs, antibodies and surgical intervention [148]. AHSCT has proved safe and can be able to induce and maintain remission in previously refractory patients affected by Crohn's disease [149,150].

By combining AHSCT with CY, a clinical remission with a disappearance of diarrhea, and a reduction in the abdominal pain and activity have been obtained [151].

#### Autoimmune cytopenias

In immune thrombocytopenia purpura (ITP), the platelets are removed from blood by autoantibodies and the effects are thrombocytopenia and bleeding. Usually, ITP cases are responsive to high doses of immunosuppressors; nevertheless this treatment exposes them to myelosuppression risks. HSCT can accelerate the reestablishment of the hematological parameters, while the number of autoimmune cells in the body decreases [152]. An American study has showed the efficacy of a combined therapy of CY and AHSCT in chronic refractory ITP treatment. The majority of patients show a long term response, suggesting that SCs can accelerate the hematological re-balance compared with classic immunotherapy [153]. A study by European Bone Marrow Transplantation (EBMT) reports the treatment of 12 cases of ITP with AHSCT. However, the responses to treatment have varied from a transient response to a continuous remission or even death related to transplantation [154]. Immune haemolytic anemia (IHA) is a hematologic disease characterized by an early destruction of erythrocytes due to an autoreaction of antibodies or complement against the membrane protein [155-157]. The few reports available do not permit to gain definitive conclusions. It has been suggested that the association between the AHSCT and immunosuppressive therapy can be an effective treatment for IHA [158]. However it has also been showed a high failure rate or even death after HSCT [159].

### Diabetes Mellitus

Type I diabetes mellitus (DM) results in a cell-mediated autoimmune attack against insulin-secreting pancreatic β-cells. Insulin regulates glucose homeostasis and, in particular, it reduces glycemia when glucose exceeds in blood. Glucose accumulation, which is typical of diabetes, damages blood vessels causing the decrease of cell perfusion. Other complications are diabetic neuropathy, consisting of a gradual loss of hand, foot and limb mobility caused by nerve degeneration, retinopathy, characterized by loss of vision and blindness for light-sensitive retina atrophy, nephropathy with a loss of removing wastes and excess water and urinary tract infection with a glucose rich urine which favours bacteria proliferation. The common therapy consists in the chronic introduction of exogenous insulin to restore glucose homeostasis, although resistance to this therapy has been observed [160-163]. SC transplantation can rehabilitate pancreatic islets and reintroduce physiological secretion of human insulin.

AHSCT improves β-cells function and frequently decreases the exogenous insulin need [20] or induces a persistent insulin independence and normal glycemic control when grafted in type 1 DM subjects [164].

Combining CY with AHSCT , an insulin-free period is achieved [22]. In particular it has been proposed a synergic action of CY and AHSCT to explain exogenous insulin independence. This has been shown in the first successful Polish attempt to achieve remission in the early phase of type 1 diabetes mellitus following immunosuppressive treatment and the subsequent AHSCT. The method involves the destruction of the patient's immune system and also the autoimmune process which is the main pathomechanism in type 1 diabetes mellitus. As soon as the autoaggressive mechanism is stopped, pancreatic cells might be able to resume secretion of sufficient amounts of insulin to maintain normal glucose level [165]. Allotropic human adipose tissue derived, insulin-making mesenchymal SCs (h-AD-MSC) have been transfused with unfractionated cultured BM in insulinopenic DM patients without side effects. Furthermore, an appreciable insulin requirement decrease has been observed [166].

### Neurological disorders

#### Amyotrophic lateral sclerosis

Amyotrophic lateral sclerosis (ASL) is caused by the progressive death of central and peripheral motor neurons. The subjects affected by ALS show a severe motor dysfunction. In several cases the mutation of the superoxide dismutase gene is inherited, but often its origin is unknown. ALS is not a typical AD because autoimmune and inflammatory abnormalities are not an etiological cause of the disease, even if they influence its progression. The therapeutic strategy, used for ALS, is intended to protect neurons from degeneration and to stimulate cell regeneration. At the moment, no drug treatment restores the neural cells. SCs therapy is a promising strategy that can combine neuroprotection with the recovery of the neuromotor function [167].

Intrathecal injection of selected HSC or MSC have resulted safe and have afforded a partial neurological function improvement in patients with severe ALS [168,169].

Ex vivo expanded AHSC spinal injection, in patients with severe impairment of the lower limb by ALS, has also showed cell number-related improvement of general condition, i.e. a deceleration of the leg muscular strength loss and a respiratory function decline. Side effects, such as intercostal pain or dysesthesia have only been slight and reversible, but they sometimes persist after 2 years from treatment [170].

AHSCT into the frontal motor cortex in ALS patients has delayed the disease progression and has improved the quality of life [171].

Many cases of ALS patients, treated with autologous SCs (mesenchymal and hematopoietic) and injection (intraspinal thoracic or in motor cortex), have been reported. A deceleration of forced vital capacity linearly declines and an improvement in functionality has been described, probably due to an immunomodulatory effect [172].

### Parkinson's disease

Parkinson's disease (PD) is a debilitating neurodegenerative disorder caused by selective and gradual loss of nigrostriatal dopamine-containing neurons [112]. Dopaminergic neurons are localized in the substantia nigra pars compacta and project on to striatum. A degeneration of these cells leads to neural circuit anomaly in the basal ganglia that regulate movement. The main symptoms are rigidity, bradykinesia, tremor and postural instability [173]. Pharmacological treatments, such as levodopa/carbidopa, dopamine agonists, MAO-B inhibitors, and COMT inhibitors, are effective to control PD symptoms but they are unable to stop neural degeneration and replace dead cells [174]. In this context SCs seem to be promising since they can stimulate the recovery of neuromotor function. PD patients, who had received unilaterally striatum human embryonic mesencephalic tissue implants twice, have showed movement improvements (different degrees) and DOPA (dopamine precursor) increased levels [175,176]. Symptoms and F-fluorodopa (marked analogous) uptake have significantly improved in PD patients younger than 60 [177].

Bilateral fetal nigral graft, in PD patients, has also resulted safe and quite effective. Fluorodopa uptake has increased, but in about half of the patients dyskinesia has remained unchanged [178,179].

#### Spinal cord lesions

Spinal trauma can break ascending and descending axonal pathways with consequent loss of neurons and glia, inflammation and demyelination. Depending on the injury site, functional effects, induced by cellular damage, are inability of movement, sensorial loss and/or lack of autonomic control. No therapies for spinal trauma exist. However, interesting results have been obtained with SCs transplantation [112].

Based on the discovery that olfactory mucosa is an important and readily disposable source of stem like progenitor cells for neural repair, the effects of its intraspinal transplant on spinal cord injured patients have been shown. All the patients have improved their motor functions either upper extremities in tetraplegics or lower extremities in paraplegics. The side effects include a transient pain, relieved with medication, and sensory decrease [180]. Generally, the olfactory mucosa transplant is safe, without tumor or persistent neuropathic pain [181]. Neurological improvements have also been observed in spinal cord injury patients treated with intra-spinal autologous BMC graft. The best results have been obtained in patients transplanted 8 weeks before the trauma [182].

### Huntington's disease

Huntington's disease (HD) is a fatal, untreated autosomal dominant characterized by CAG trinucleotide repeats located in the Huntington's gene. This neurodegenerative disorder is characterized by chorea, i.e. excessive spontaneous movements and progressive dementia. The death of the neurons of the corpus striatum causes the main symptoms [112]. At the moment, no therapies for HD exist although SCs can contrast the neurodegeneration characteristic of the disease. In a HD patient, who died 18 months after human fetal striatal tissue transplantation for a cardiovascular disease, postmortem histological analysis has showed the survival of the donor's cells. No histological evidence of rejection has been observed. The donor's fetal neural cells do not have mutated huntingtin aggregate and currently are supposed to be able to replace the damaged host neurons and reconstitute the damaged neuronal connections [183].

Several studies have emphasized safety [184,185], the donor's cells survival [183] and the functional efficacy [186,187] of intracerebral fetal striatal transplantation practice.

However, three cases of post-graft subdural hematomas, in late-stage HD patients, have been reported. The same authors have observed that striatal graft, in heavily atrophied basal ganglia, probably increases hematoma risk [188].

#### Stroke

The obstruction of a cerebral artery leads to focal ischemia, loss of neurons and glial cells with the consequent motor, sensory or cognitive impairments. Recent advances in thrombolysis and in neuroprotective strategies allow managing acute stroke. When drugs are administered few minutes after the injury and the damage is not severe, it is possible to restore the normal functions [112]. Interesting results are also obtained with the SC therapy.

A subarachnoidal injection of immature nervous cells and hematopoietic tissue suspension, in patients with brain stroke, have significantly improved the functional activity without serious side effects [189].

Progressively, neurological deficits have decreased in cerebral infracted patients, when treated with intravenous MSCs infusion. No adverse cell-related, serological or imaging defined effects have been observed [190].

Interesting results have been obtained with the granulocyte colony-stimulating factor (G-CSF) in the acute cerebral infarction management. G-CSF has mobilized HSCs, improving the metabolic activity and the neurologic outcomes [191].

#### Duchenne muscular dystrophy

Duchenne muscular dystrophy **(**DMD) is a severe recessive X-linked muscular dystrophy characterized by progressive muscle degeneration, loss in ambulation, paralysis, and finally death. DMD is caused by mutations on the DMD gene, located on the X chromosome. DMD symptoms are principally musculoskeletal, i.e. muscle fiber and skeletal deformities, difficulties in motor skills and fatigue, but they can regard one's behavior and learning. To date, no cures for DMD are known, while treatments, such as corticosteroids, physical therapy and orthopedics appliance can control the symptoms to maximize the quality of life [192]. Recent developments in SC research suggest the possibility to replace the damaged muscle tissue.

Allogenic, combined with CY, or autologous myoblast transplantation in DMD patients is a safe procedure. No local or systemic side effects have been reported [193,194]. In particular, using fluorescence in situ hybridization (FISH), myoblast allograft has showed the donor's nuclei fused with the host's nuclei and dystrophin wild type increased [195]. Therefore distrophin mRNA has been detected using polymerase chain reaction (PCR), six months after graft [196]. However, many authors have reported that myoblast injection in DMD patients do not improve their strength [194], even if the injection site, CY dose or blast number have changed [196,197]. An injection-triggered cellular immune response in the host has been discovered. The antibodies producted are capable to fix the complement and destroy new myotubes. Probably distrophin is an antigen recognized by the host immune system [198].

#### Heart failure

Heart failure is commonly caused by myocardial infarction (MI). MI is the ischemic necrosis of the cardiac tissue and it is frequently triggered by severe coronary stenosis. The myocyte fall produces abnormal left-ventricular remodelling the chamber dilatation and contractile dysfunction [199]. The rapid reperfusion of the infarct related coronary artery is the primary management to reduce the ischemic area and avoid the myocardic tissue damage. The percutaneous transluminal coronary angioplasty, with a stent implantation, is the gold standard method to reestablish the coronary flow. Unfortunately, angioplasty is effective only if executed rapidly and expertly, otherwise the myocardial necrosis, which starts several minutes after the coronary occlusion, commits the cardiac function [200]. Many studies suggest that SCs can improve heart function by repairing the cardiac tissue.

The major multicenter trial on MI treatment with autologous skeletal myoblast transplantation, has reported the failure of cell therapy in heart dysfunction. No improvements in the echocardiographic heart function have been underlined, neither general health has taken a turn for the worse [201]. However, other studies have described the efficacy of myoblast transplant in the ejection fraction (EF) improvement in MI patients [202,203].

Instead, AHSCT improves cardiovascular conditions in MI patients, such as ejection fraction, and it avoids harmful left ventricular remodelling [204].

In particular, intracoronary infusion of HSCs is associated with a significant reduction of the occurrence of major adverse cardiovascular events after MI, such as MI recurrence restenosis or arrhythmia [205,206].

### Ocular surface diseases

Ocular surface diseases are characterized by persistent epithelial defects, corneal perfusion problems, chronic inflammation, scarring and conjunctivalisation resulting in visual loss. These pathologies are associated with a limbal SC deficiency (LSCD). LSCD derives from hereditary disorders, such as aniridia, keratitis, or acquired disorders, such as Stevenson-Johnson syndrome (SJS), chemical injuries, ocular cicatricial pemphigoid, contact lens-induced keratopathy, multiple surgery or limbal region cryotherapy , neurotrophic keratopathy and peripheral ulcerative keratitis conditions [207]. Obviously, SC transplantation is the only effective therapy that can restore the ocular environment.

A study conducted on a homogeneous group of patients with limbal cell deficiency has been conducted using SCs obtained from the limbus of the contralateral eye. Fibrin cultures were grafted onto damaged corneas observing that: 1) fibrin-cultured limbal SCs were successful in 14 of 18 patients; 2) re-epithelialization occurred within the first week; 3) inflammation and vascularization regressed within the first 3-4 weeks; 4) by the first month, the corneal surface was covered by a transparent, normal-looking epithelium; 4) at 12-27 months follow-up, corneal surfaces were clinically and cytologically stable. Their visual acuity improved from light perception or counting fingers to 0.8-1.0 [208]. Limbal allograft also corrects acquired and hereditary LSCD recovering the visual activity [209-211]. It has been reported a retrospective study on endothelial rejection in central penetrating graft after a simultaneous keratolimbal allograft transplantation (KLAT) and penetrating keratoplasty (PKP) using the same donor's cornea. A third cohort of treated patients have rejected transplant. After an immunosuppressive therapy, the majority of rejects have restored the corneal clarity while in the others neovascularization has developed into the grafted limbs [212].

#### Cartilage repair

Osteoarthritis (OA) is a degenerative joint disease, characterized by accumulated mechanical stresses to joints and leading to the destruction of articular cartilage. A synovial fluid decrease has also been observed [213]. OA and peripheral joint injuries are commonly treated with interventional pain practice, exercise therapy, ultrasound or electromagnetic device after surgery, although these therapies have not proven to be a definitive solution [214-217]. SCs seem to be a promising solution to overcome OA cartilage destruction. The first autologous mesenchymal SC culture and percutaneous injection into a knee with symptomatic and radiographic degenerative joint disease has been reported and it has resulted in significant cartilage growth, decreased pain and increased joint mobility. This has significant future implications for minimally invasive treatment of osteoarthritis and meniscal injury treated with percutaneous injection of autologous MSCs expanded ex-vivo has been reported [218].

### Liver disease

Cirrhosis is a progressive liver function loss caused by fibrous scar tissue replacement of normal parenchyma. Cirrhosis is commonly caused by alcoholism, hepatitis B and C and fatty liver disease, but there are many other possible causes. Cirrhosis is generally irreversible and treatments are generally focused on preventing its progression and complications. Only liver transplant can revert the pathological condition if there is a terminally ill patient [219]. SC therapy can contrast liver degeneration and block cirrhosis progression.

AHSC infusion in cirrhotic patients has improved liver parameters, such as transaminase, bilirubin decrease and albumin increase [220,221]. After infusion, proliferation indexes, such as alpha fetoprotein and proliferating cell nuclear antigen (PCNA), have significantly increased, suggesting that HSCs can enhance and accelerate hepatic regeneration [222]. No significant side effects have been registered [223].

## Cancer

### Renal cell cancer

Renal cell cancer (RCC) is the most frequent kidney cancer. RCC originates in the lining of the proximal convoluted renal tubule. RCC appears as a yellowish, multilobulated tumor in the renal cortex, which frequently contains zones of necrosis, hemorrhage and scarring. The signs may include blood in the urine, loin pain, abdominal mass, anaemia, varicocele, vision abnormalities, pallor, hirsutism, constipation, hypertension, hypercalcemia, night sweats and severe weight loss. The initial treatment is commonly a radical or partial nephrectomy. Other treatment strategies, including hormone therapy, chemotherapy, and immunotherapy, have little impact on global survival [224,225]. HSCT can be an important tool for the management of RCC, in particular under the metastatic form.

HSCT, combined with the immunosuppressive or donor's lymphocyte infusion (DLI), can improve the general condition in metastatic RCC patients. Three factors, i.e. performance status, C-reactive protein (CRP) level and lactate dehydrogenase (LDH) level, have been found and they are significantly associated with a major success of allograft [226]. HSCT have trigged graft versus tumor (GVT) response, reducing the metastasis and reaching out the survival time [227-229].

### Breast cancer

Breast cancer (BR) refers to cancers originating from the breast tissue, commonly from the inner lining of milk ducts or the lobules that supply the ducts with milk. Occasionally, BR presents as a metastatic disease with spreads in bones, liver, brain and lungs. The first evidence or subjective sign of BR is typically a lump that feels different from the rest of the breast tissue. Other symptoms can be: changes in breast size or shape, skin dimpling, nipple inversion, or spontaneous single-nipple discharge. Pain ("mastodynia") is an unreliable tool to determine the presence or absence of BR, but it may be indicative of other breast health issues. When the cancer cells invade the dermal lymphatics (small lymph vessels) in the breast skin, BR appears as a cutaneous inflammation. In this phase symptoms include pain, swelling, warmth and redness throughout the breast, as well as an orange peel texture to the skin, referred to as "peau d'orange". Treatment includes surgery, drugs (hormonal therapy and chemotherapy), and radiation, which are effective against non metastatic forms [230]. SCT can increase survival in patients with spreading BR.

A high dose chemotherapy (HDC) with SC support has improved the disease free survival in metastatic BR. However, HDC has induced serious cytotoxicities [231]. In reduced intensity conditioning regimens (RICT), allogeneic HSCT has proven to be effective in persistent and progressive metastatic BR, decreasing relapse. Allogeneic SC transplantation with myeloablative conditioning regimens may provide cytoreduction and eradication of disease with a cancer free-graft and an immune-mediated graft-versus-tumor (GVT) effect mediated by the donor's immune cells [232,233].

### Colorectal cancer

Colorectal cancer (CRC) includes cancerous growths in the colon, rectum and appendix. Many CRCs are thought to arise from adenomatous polyps in the colon. These mushroom like growths are usually benign, but some may develop into cancer over time. Symptoms and signs are divided into: local ones, consisting in change in bowel habits and in frequency, such as constipation and/or diarrhea, feeling of incomplete defecation (tenesmus) and reduction in tool diameter, bloody stools or rectal bleeding, stools with mucus, black and tar-like stool (melena), bowel pain, bloating and vomiting, hematuria or pneumaturia, or smelly vaginal discharge; constitutional ones i.e. weight loss, anemia, dizziness, fatigue and palpitations; metastatic ones, i.e. liver metastases, causing Jaundice, pain in the abdomen, liver enlargement and blood clots in veins and arteries. Surgery is the usual therapy and, in many cases, is followed by chemotherapy [234-236]. The gastrointestinal tract is a target of GVHD in transplants and, therefore, CRC, might be treated by allogeneic SCT. Four cases of metastatic CRC, undergoing reduced-intensity SC transplantation (RIST), have been reported. No significant graft toxicities have been registered. CRC markers have decreased in three patients after allograft. Three patients died of disease progression, but postmortem examination has showed a macroscopic metastatic lesion disappearance [237]. The patients with progressing metastatic CRC, treated with RIST, have showed relevant results in terms of tumor response. Even metastatic CRC need intense GVT to eradicate spreading tumor cells. Allogeneic SCT is likely to have trigged the generation of anti-neoplastic T cells [238-240].

### Ovarian cancer

Ovarian cancer (OC) is a cancerous growth arising from different parts of the ovary. Commonly, OC arises from the outer lining of the ovary, but also from the Fallopian tube or egg cells. OC is characterized by non-specific symptoms and, in early stages, it is associated with abdominal distension. Many women with OC report one or more non-specific symptoms, such as an abdominal pain or discomfort, an abdominal mass, bloating, back pain, urinary urgency, constipation, tiredness, and some specific symptoms, such as pelvic pain, abnormal vaginal bleeding or involuntary weight loss. There can be a build-up of fluid (ascites) in the abdominal cavity. A surgical treatment may be sufficient for malignant tumors that are well-differentiated and confined to the ovary. An addition of chemotherapy may be required for the most aggressive tumors that are confined to the ovary. For patients with an advanced disease, a surgical reduction is combined with a standard chemotherapy regimen. Some studies describe the feasibility of the combination of chemotherapy with SCT [241].

Allogeneic HSCT, associated with chemotherapy in advanced OC, treatment has induced variable effects. When SCs infusion trigger GVT, it is possible to control the disease progression [242,243]. However, GVT does not occur frequently. No serious side effects have been registered [244,245].

### Lung cancer (LC)

LC is characterized by an uncontrolled cell growth in the lung tissue. Frequently LC rises from the epithelial cells. The small cell lung carcinoma (SCLC) is the most frequent lung carcinoma. The symptoms can result from the local growth of the tumor (coughing up blood, shortness of breath and chest pain), a spread to the nearby areas (hoarseness of voice, shortness of breath, difficulty in swallowing, swelling of the face and hands), a distant spread (the spread to the brain can cause headache, blurring of vision, nausea, vomiting, and weakness of any limb, a spread to the vertebral column which can cause back pain, a spread to the spinal cord which can cause paralysis, a spread to the bone that may lead to bone pain and a spread to the liver possibly causing pain in the right upper part of the abdomen), paraneoplastic syndromes, or a combination of them. Possible treatments are surgery, chemotherapy, and radiotherapy [246]. An addition of SCT can improve the survival rate and avoid relapses. AHSCT has been frequently combined with chemotherapy in SCLC treatment. The reason is that HSCs drastically reduce the chemotherapy side effects, in particular myeloablation [247-249]. Probably, HSCs may also induce therapeutic effects contrasting the tumor directly [250]. In SCLC, HSCs trigger GVT and increase the survival rate.

### Leukemia

Leukemia is the uncontrolled proliferation of the myeloid or lymphoid blood line and the consequential blast accumulation in the BM. Leukemia can be classified in acute myeloid leukemia (AML), chronic myeloid leukemia (CML), acute lymphoblastic leukemia (ALL) and chronic lymphocytic leukemia (CLL). Leukemia is caused by a mutation in the gene involved in the cell proliferation. The first signs and symptoms of leukemia are nonspecific and they include fatigue, malaise, and abnormal bleeding, excessive bruising, weakness, reduced exercise tolerance, weight loss, bone or joint pain, infection and fever, abdominal pain or "fullness", enlarged spleen, lymph nodes and liver,. Moreover a high white blood cell count is detectable. Chemotherapy is the initial treatment of choice, but only with the substitution of the malignant blast with the normal SCs, leukemia can be eradicated [251-256].

Many studies indicate allogenic RIST as an important procedure to achieve a complete remission in patients with leukemia, especially if a human leukocyte antigen compatible donor is employed [257-265]. GVHD is the major limiting factor for successful transplantation, but its frequency is sensibly reduced if compared to the first treatment [266,267]. The mortality rate has also decreased significantly [268].

## Guidelines For Scs Application

SCs transplantation in human patients must ensure safety and therapeutic efficacy. Preclinical studies aim at providing persuasive evidence, in an appropriate in vitro and/or animal model, which supports the likelihood of a relevant positive clinical outcome. Preclinical testing in animal models, whenever feasible, is especially important for SC based approaches because SCs can act through multiple mechanisms. Physiological integration and long-lived tissue reconstitution are hallmarks of SC based therapeutics for many disease applications. Animal models will be important to assess possible adverse effects of implanted cellular products. The need for animal model is especially strong in the case of extensive ex vivo manipulation of cells and/or when the cells have been derived from pluripotent SCs.

It should be acknowledged, however, that preclinical assays, including studies in animal models, may provide limited insight into how transplanted human cells will behave in human recipients due to the context dependent nature of the cell behavior and recipient's immune response. These uncertainties must be borne in mind during the independent peer review of the preclinical data. Only when the compelling preclinical data are available, careful and incremental testing in patients is justified. Preclinical studies must be subject to rigorous and independent peer review and regulatory oversight prior to the initiation of the clinical trials, in order to ensure that the performance of the clinical studies is scientifically and medically warranted. Because new and unforeseen safety concerns may arise with the clinical translation, frequent interaction, between preclinical and clinical investigators, is strongly encouraged. The clinical trials of SC based interventions must follow internationally accepted principles governing the ethical conduct of the clinical research and the protection of the human subjects. Key requirements include regulatory oversight, peer review by an expert panel independent of the investigators and sponsors, fair subject selection, informed consent and patient monitoring. However, there is a number of important SC related issues that merit a special attention [269]. The guidelines concerning the preclinical studies (animal model), clinical studies have been summarized in the "Guidelines for the Clinical Translation of Stem Cells" published in 2008.

## Conclusions

This review shows the most interesting clinical trials in SC biology and regenerative medicine [270-272]. Promising results have been described in disorders, such as diabetes [273] and neurodegenerative diseases [274,275], where SCs graft can reestablish one or more deficit cellular lineages and, generally, a healthy state. Notably, many clinical studies have underlined the immunomodulatory effect of SCs in autoimmune diseases, such as multiple sclerosis [275], organ transplants [276] and in uncontrolled immune-inflammatory reactions [277-279]. Probably, SCs induce immune suppression and inhibit proliferation of alloreactive T cells [280]. Moreover, SCs are at the core of the huge framework of cellular therapy and are going to be used in the gene therapy [281,282] or as scaffolds in SCNT [109]. An interesting cell type is the induced pluripotent stem cell (iPSC) [283]. iPSCs are artificial cells derived from non pluripotent cells, typically adult somatic cells through the induction of a "forced" expression of specific genes.

iPSCs have been regarded as the most promising way to create SCs. However the use of iPSCs has raised concerns. The iPSCs are easily created by modulating the human genome to ectopically express transcriptional factors. Since their overexpression has been associated with tumorigenesis [284,285], there is a risk that the differentiated cells might also be tumorigenic when transplanted into patients. The insertion of transgenes into functional genes of the human genome can be detrimental [286]. Furthermore, although the transcription factors are mostly silenced following reprogramming, it has been reported that residual transgene expression may be responsible for some of the differences between ESCs and iPSCs such as the altered differentiation potential of iPSCs into functional cell types [287]. There are a few ways of creating iPSCs, i.e. genomic modification, protein introduction, and treatment with chemical reagents [288,289]. iPSCs research has to be conducted keeping in mind ethical, legal, and social issues [290].

These cells may be used to construct disease models and to screen effective and safe drugs, as well as to treat patients through the cell transplantation therapy [281]. However, the validity of these predictions will depend on the benefits obtained on the ongoing phase II and III human clinical trials. In the meantime, new candidate small molecules and bioactives will be identified using SC assays in the high-throughput screening that will impact on SC mobilization broaden the horizons of regenerative medicine. It has been proposed that centenarians and supercentenarians (aged 110 years or more) may present an unprecedented opportunity to explore the possibilities of SCs that have proven their value over time. These SCs should be studied to determine their developmental potential, mutational load, telomere lengths, and markers of "stemness" [291]. In conclusion, beyond the great enthusiasm for new treatment perspectives, an heavy investigational work is still in progress to develop specific SCs related pharmacology. In fact new drugs are urgently needed to assist SCs in vitro/in vivo differentiation and full tissue/organ integration and recovery. As far as CNS related diseases (cerebrovascular accidents and spinal traumatic lesions) are concerned, the role of autologous cytokines induced by SCs infusion has to be deeply investigated and may represent, in the future, a new treatment perspective.

## Abbreviations

(ADSC): Adipose Tissue-Derived Stromal Cell; (ASC): Adult Stem Cell; (ALL): Acute Lymphoblastic Leukemia; (AML): Acute Myeloid Leukemia; (ASL): Amyotrophic Lateral Sclerosis; (AD): Autoimmune Diseases; (AHSCT): Autologous HSCT; (BM): Bone Marrow; (BR): Breast Cancer; (BOOP): Bronchiolitis Obliterans Organizing Pneumonia; (CNS): Central Nervous System; (CML): Chronic Myeloid Leukemia; (CLL): Chronic Lymphocytic Leukemia; (CRC): Colorectal Cancer; (CRP): C-Reactive Protein; (CY): Cyclophosphamide; (DM): Diabetes Mellitus; (DMARD): Disease-Modifying Anti-Rheumatic Drug; (DLI): Donor Lymphocyte Infusion; (DMD): Duchenne Muscular Dystrophy; (EF): Ejection Fraction; (EC): Embryonic cell; (EGC): Embryonic Germ Cell; (ESC): Embryonic Stem Cell; (EBMT): European Bone Marrow Transplantation; (FSC): Fetal Stem Cell; (GVHD): Graft Versus Host Disease; (GVT): Graft Versus Tumor; (HSC): Haematopoietic Stem Cell; (HDC): High-Dose Chemotherapy; (HSCT): HSC Transplantation; (h-AD-MSC): Human Adipose-Tissue-Derived insulin-making Mesenchymal SCs; (HD): Huntington's Disease; (IHA): Immune Haemolytic Anemia; (ITP): Immune Thrombocytopenia Purpura; (iPSC): Induced Pluripotent Stem Cell; (ICM): Inner Cell Mass; (JIA): Juvenile Idiopathic Arthritis; (KLAT): Keratolimbal Allograft Transplantation; (LDH): Lactate Dehydrogenase; (LONIPC): Late-Onset Non-Infectious Pulmonary Complications; (LSCD): Limbal SC Deficiency; (LC): Lung Cancer; (MHC): Major Histocompatability Complex; (MSC): Mesenchimal Stem Cell; (MS): Multiple Sclerosis; (MI): Myocardial Infarction; (OB): Obliterative Bronchiolitis; (OA): Osteoarthritis; (OC): Ovarian Cancer; (PD): Parkinson's Disease; (PCR): Polymerase Chain Reaction; (PPMS): Primary Progressive MS; (PCNA): Proliferating Cell Nuclear Antigen; (RIST): Reduced-Intensity Stem-Cell Transplantation; (RICT): Reduced-Intensity Conditioning Regimens; (REB): Research Ethics Board; (RA): Rheumatoid Arthritis; (RCC): Renal Cell Cancer; (SPMS): Secondary Progressive MS; (SCNT): Somatic Cell Nuclear Transfer; (SCLC): Small Cell Lung Carcinoma; (SC): Stem Cell; (SCOC): Stem Cell Oversight Committee; (SF): Synovial Fluid; (SLE): Systemic Lupus Erythematosus; (SSc): Systemic Sclerosis; (UCE): Umbilical Cord Epithelium; (UCB): Umbilical Cord Blood; (UC-HS): Umbilical Cord Hematopoietic; (UC-MS): Umbilical Cord Mesenchymal; (VOD): Veno-Occlusive Disease;

## Competing interests

The authors declare that they have no competing interests.

## Authors' contributions

The authors, namely DL, TI and BP, contributed equally to this work. All authors read and approved the final manuscript.

## Aknowledgements

This review was not supported by grants. The authors hereby certify that all work contained in this review is original work of DL, TI and BP. All the information taken from other articles, including tables and pictures, have been referenced in the "Bibliography" section. The authors claim full responsibility for the contents of the article.

## References

[B1] Pittenger MF Mackay AM Beck SC Jaiswal RK Douglas R Mosca JD Moorman MA Simonetti DW Craig S Marshak DR Multilineage potential of adult human mesenchymal stem cells Science 1999 284 5411 143 147 10.1126/science.284.5411.143 10102814

[B2] Mayhall EA Paffett-Lugassy N Zon LI The clinical potential of stem cells Curr Opin Cell Biol 2004 16 6 713 720 10.1016/j.ceb.2004.09.007 15530786

[B3] Fortier LA Stem cells: classifications, controversies, and clinical applications Vet Surg 2005 34 5 415 423 10.1111/j.1532-950X.2005.00063.x 16266332

[B4] Zhong W Timing cell-fate determination during asymmetric cell divisions Curr Opin Neurobiol 2008 18 5 472 478 10.1016/j.conb.2008.10.005 18983918 PMC2609754

[B5] Doe CQ Neural stem cells: balancing self-renewal with differentiation Development 2008 135 9 1575 1587 10.1242/dev.014977 18356248

[B6] Knoblich JA Mechanisms of asymmetric stem cell division Cell 2008 132 4 583 597 10.1016/j.cell.2008.02.007 18295577

[B7] Zhong W Chia W Neurogenesis and asymmetric cell division Curr Opin Neurobiol 2008 18 1 4 11 10.1016/j.conb.2008.05.002 18513950

[B8] Baksh D Song L Tuan RS Adult mesenchymal stem cells: characterization, differentiation, and application in cell and gene therapy J Cell Mol Med 2004 8 3 301 316 10.1111/j.1582-4934.2004.tb00320.x 15491506 PMC6740223

[B9] Barry FP Murphy JM Mesenchymal stem cells: clinical applications and biological characterization Int J Biochem Cell Biol 2004 36 4 568 584 10.1016/j.biocel.2003.11.001 15010324

[B10] Thomson JA Itskovitz-Eldor J Shapiro SS Waknitz MA Swiergiel JJ Marshall VS Jones JM Embryonic stem cell lines derived from human blastocysts Science 1998 282 5391 1145 1147 10.1126/science.282.5391.1145 9804556

[B11] Baharvand H Ashtiani SK Valojerdi MR Shahverdi A Taee A Sabour D Establishment and in vitro differentiation of a new embryonic stem cell line from human blastocyst Differentiation 2004 72 5 224 229 10.1111/j.1432-0436.2004.07205005.x 15270778

[B12] Ladurner P Rieger R Baguna J Spatial distribution and differentiation potential of stem cells in hatchlings and adults in the marine platyhelminth macrostomum sp.: a bromodeoxyuridine analysis Dev Biol 2000 226 2 231 241 10.1006/dbio.2000.9867 11023683

[B13] Fang TC Alison MR Wright NA Poulsom R Adult stem cell plasticity: will engineered tissues be rejected? Int J Exp Pathol 2004 85 3 115 124 10.1111/j.0959-9673.2004.00380.x 15255965 PMC2517466

[B14] Pera MF Reubinoff B Trounson A Human embryonic stem cells J Cell Sci 2000 113 Pt 1 5 10 10591620 10.1242/jcs.113.1.5

[B15] Pessina A Gribaldo L The key role of adult stem cells: therapeutic perspectives Curr Med Res Opin 2006 22 11 2287 2300 10.1185/030079906X148517 17076989

[B16] Gucciardo L Lories R Ochsenbein-Kolble N Done E Zwijsen A Deprest J Fetal mesenchymal stem cells: isolation, properties and potential use in perinatology and regenerative medicine BJOG 2009 116 2 166 172 10.1111/j.1471-0528.2008.02005.x 19076948

[B17] Blau HM Brazelton TR Weimann JM The evolving concept of a stem cell: entity or function? Cell 2001 105 7 829 841 10.1016/S0092-8674(01)00409-3 11439179

[B18] Joshi D Behari M Neuronal stem cells Neurol India 2003 51 3 323 328 14652430

[B19] Fan J Varshney RR Ren L Cai D Wang DA Synovium-derived mesenchymal stem cells: a new cell source for musculoskeletal regeneration Tissue Eng Part B Rev 2009 15 1 75 86 10.1089/ten.teb.2008.0586 19196118

[B20] Voltarelli JC Couri CE Stracieri AB Oliveira MC Moraes DA Pieroni F Coutinho M Malmegrim KC Foss-Freitas MC Simoes BP Autologous nonmyeloablative hematopoietic stem cell transplantation in newly diagnosed type 1 diabetes mellitus JAMA 2007 297 14 1568 1576 10.1001/jama.297.14.1568 17426276

[B21] Sangwan VS Fernandes M Bansal AK Vemuganti GK Rao GN Early results of penetrating keratoplasty following limbal stem cell transplantation Indian J Ophthalmol 2005 53 1 31 35 10.4103/0301-4738.15282 15829744

[B22] Rosa SB Voltarelli JC Chies JA Pranke P The use of stem cells for the treatment of autoimmune diseases Braz J Med Biol Res 2007 40 12 1579 1597 17713674 10.1590/s0100-879x2006005000166

[B23] Kallis YN Alison MR Forbes SJ Bone marrow stem cells and liver disease Gut 2007 56 5 716 724 10.1136/gut.2006.098442 17145739 PMC1942133

[B24] Varanou A Page CP Minger SL Human embryonic stem cells and lung regeneration Br J Pharmacol 2008 155 3 316 325 10.1038/bjp.2008.333 18724383 PMC2567888

[B25] Nieto Y Jones RB Shpall EJ Stem-cell transplantation for the treatment of advanced solid tumors Springer Semin Immunopathol 2004 26 1-2 31 56 10.1007/s00281-004-0160-8 15368078

[B26] Filip S Mokry J Horacek J English D Stem cells and the phenomena of plasticity and diversity: a limiting property of carcinogenesis Stem Cells Dev 2008 17 6 1031 1038 10.1089/scd.2007.0234 19006453

[B27] Vicente-Duenas C Gutierrez de Diego J Rodriguez FD Jimenez R Cobaleda C The role of cellular plasticity in cancer development Curr Med Chem 2009 16 28 3676 3685 10.2174/092986709789105019 19747147

[B28] Reddy P Arora M Guimond M Mackall CL GVHD: a continuing barrier to the safety of allogeneic transplantation Biol Blood Marrow Transplant 2009 15 1 Suppl 162 168 10.1016/j.bbmt.2008.10.014 19147099 PMC2633357

[B29] Zarzeczny A Caulfield T Emerging ethical, legal and social issues associated with stem cell research & and the current role of the moral status of the embryo Stem Cell Rev 2009 5 2 96 101 10.1007/s12015-009-9062-4 19521800

[B30] Wobus AM Boheler KR Embryonic stem cells: prospects for developmental biology and cell therapy Physiol Rev 2005 85 2 635 678 10.1152/physrev.00054.2003 15788707

[B31] Oligny LL Human molecular embryogenesis: an overview Pediatr Dev Pathol 2001 4 4 324 343 10.1007/s10024001-0033-2 11441334

[B32] Talbot NC Powell AM Rexroad CE Jr In vitro pluripotency of epiblasts derived from bovine blastocysts Mol Reprod Dev 1995 42 1 35 52 10.1002/mrd.1080420106 8562049

[B33] Odorico JS Kaufman DS Thomson JA Multilineage differentiation from human embryonic stem cell lines Stem Cells 2001 19 3 193 204 10.1634/stemcells.19-3-193 11359944

[B34] Sjogren A Hardarson T Andersson K Caisander G Lundquist M Wikland M Semb H Hamberger L Human blastocysts for the development of embryonic stem cells Reprod Biomed Online 2004 9 3 326 329 10.1016/S1472-6483(10)62149-9 15353085

[B35] Cowan CA Klimanskaya I McMahon J Atienza J Witmyer J Zucker JP Wang S Morton CC McMahon AP Powers D Derivation of embryonic stem-cell lines from human blastocysts N Engl J Med 2004 350 13 1353 1356 10.1056/NEJMsr040330 14999088

[B36] Rosler ES Fisk GJ Ares X Irving J Miura T Rao MS Carpenter MK Long-term culture of human embryonic stem cells in feeder-free conditions Dev Dyn 2004 229 2 259 274 10.1002/dvdy.10430 14745951

[B37] Fujioka T Yasuchika K Nakamura Y Nakatsuji N Suemori H A simple and efficient cryopreservation method for primate embryonic stem cells Int J Dev Biol 2004 48 10 1149 1154 10.1387/ijdb.041852tf 15602701

[B38] Zhou CQ Mai QY Li T Zhuang GL Cryopreservation of human embryonic stem cells by vitrification Chin Med J (Engl) 2004 117 7 1050 1055 15265381

[B39] Reubinoff BE Pera MF Vajta G Trounson AO Effective cryopreservation of human embryonic stem cells by the open pulled straw vitrification method Hum Reprod 2001 16 10 2187 2194 10.1093/humrep/16.10.2187 11574514

[B40] Hayflick L Moorhead PS The serial cultivation of human diploid cell strains Exp Cell Res 1961 25 585 621 10.1016/0014-4827(61)90192-6 13905658

[B41] Campisi J From cells to organisms: can we learn about aging from cells in culture? Exp Gerontol 2001 36 4-6 607 618 10.1016/S0531-5565(00)00230-8 11295503

[B42] Wright WE Shay JW Historical claims and current interpretations of replicative aging Nat Biotechnol 2002 20 7 682 688 10.1038/nbt0702-682 12089552

[B43] D'Ippolito G Schiller PC Ricordi C Roos BA Howard GA Age-related osteogenic potential of mesenchymal stromal stem cells from human vertebral bone marrow J Bone Miner Res 1999 14 7 1115 1122 10404011 10.1359/jbmr.1999.14.7.1115

[B44] Brook FA Gardner RL The origin and efficient derivation of embryonic stem cells in the mouse Proc Natl Acad Sci USA 1997 94 11 5709 5712 10.1073/pnas.94.11.5709 9159137 PMC20843

[B45] O'Donoghue K Fisk NM Fetal stem cells Best Pract Res Clin Obstet Gynaecol 2004 18 6 853 875 15582543 10.1016/j.bpobgyn.2004.06.010

[B46] Gallacher L Murdoch B Wu D Karanu F Fellows F Bhatia M Identification of novel circulating human embryonic blood stem cells Blood 2000 96 5 1740 1747 10961872

[B47] Fortier LA Nixon AJ Williams J Cable CS Isolation and chondrocytic differentiation of equine bone marrow-derived mesenchymal stem cells Am J Vet Res 1998 59 9 1182 1187 9736400

[B48] Deasy BM Li Y Huard J Tissue engineering with muscle-derived stem cells Curr Opin Biotechnol 2004 15 5 419 423 10.1016/j.copbio.2004.08.004 15464371

[B49] Zuk PA Zhu M Mizuno H Huang J Futrell JW Katz AJ Benhaim P Lorenz HP Hedrick MH Multilineage cells from human adipose tissue: implications for cell-based therapies Tissue Eng 2001 7 2 211 228 10.1089/107632701300062859 11304456

[B50] De Bari C Dell'Accio F Tylzanowski P Luyten FP Multipotent mesenchymal stem cells from adult human synovial membrane Arthritis Rheum 2001 44 8 1928 1942 10.1002/1529-0131(200108)44:8<1928::AID-ART331>3.0.CO;2-P 11508446

[B51] Zarnett R Salter RB Periosteal neochondrogenesis for biologically resurfacing joints: its cellular origin Can J Surg 1989 32 3 171 174 2713771

[B52] Wong MH Regulation of intestinal stem cells J Investig Dermatol Symp Proc 2004 9 3 224 228 10.1111/j.1087-0024.2004.09304.x 15369217

[B53] Blanpain C Lowry WE Geoghegan A Polak L Fuchs E Self-renewal, multipotency, and the existence of two cell populations within an epithelial stem cell niche Cell 2004 118 5 635 648 10.1016/j.cell.2004.08.012 15339667

[B54] Miura M Gronthos S Zhao M Lu B Fisher LW Robey PG Shi S SHED: stem cells from human exfoliated deciduous teeth Proc Natl Acad Sci USA 2003 100 10 5807 5812 10.1073/pnas.0937635100 12716973 PMC156282

[B55] McKay RD Stem cell biology and neurodegenerative disease Philos Trans R Soc Lond B Biol Sci 2004 359 1445 851 856 10.1098/rstb.2004.1472 15293812 PMC1693367

[B56] Young HE Ceballos EM Smith JC Mancini ML Wright RP Ragan BL Bushell I Lucas PA Pluripotent mesenchymal stem cells reside within avian connective tissue matrices In Vitro Cell Dev Biol Anim 1993 29A 9 723 736 10.1007/BF02631429 8407716

[B57] Smart N Riley PR The stem cell movement Circ Res 2008 102 10 1155 1168 10.1161/CIRCRESAHA.108.175158 18497316

[B58] Behrstock S Ebert AD Klein S Schmitt M Moore JM Svendsen CN Lesion-induced increase in survival and migration of human neural progenitor cells releasing GDNF Cell Transplant 2008 17 7 753 762 10.3727/096368908786516819 19044202 PMC2650839

[B59] Wognum AW Eaves AC Thomas TE Identification and isolation of hematopoietic stem cells Arch Med Res 2003 34 6 461 475 10.1016/j.arcmed.2003.09.008 14734086

[B60] van Bekkum DW Bone marrow transplantation Transplant Proc 1977 9 1 147 154 17188

[B61] Mimeault M Batra SK Recent progress on tissue-resident adult stem cell biology and their therapeutic implications Stem Cell Rev 2008 4 1 27 49 10.1007/s12015-008-9008-2 18288619 PMC3828649

[B62] Chen FH Rousche KT Tuan RS Technology Insight: adult stem cells in cartilage regeneration and tissue engineering Nat Clin Pract Rheumatol 2006 2 7 373 382 10.1038/ncprheum0216 16932723

[B63] Bianco P Robey PG Simmons PJ Mesenchymal stem cells: revisiting history, concepts, and assays Cell Stem Cell 2008 2 4 313 319 10.1016/j.stem.2008.03.002 18397751 PMC2613570

[B64] Menicanin D Bartold PM Zannettino AC Gronthos S Genomic profiling of mesenchymal stem cells Stem Cell Rev 2009 5 1 36 50 10.1007/s12015-009-9056-2 19224407

[B65] Alison MR Poulsom R Jeffery R Dhillon AP Quaglia A Jacob J Novelli M Prentice G Williamson J Wright NA Hepatocytes from non-hepatic adult stem cells Nature 2000 406 6793 257 10.1038/35018642 10917519

[B66] Ortiz LA Gambelli F McBride C Gaupp D Baddoo M Kaminski N Phinney DG Mesenchymal stem cell engraftment in lung is enhanced in response to bleomycin exposure and ameliorates its fibrotic effects Proc Natl Acad Sci USA 2003 100 14 8407 8411 10.1073/pnas.1432929100 12815096 PMC166242

[B67] Brazelton TR Rossi FM Keshet GI Blau HM From marrow to brain: expression of neuronal phenotypes in adult mice Science 2000 290 5497 1775 1779 10.1126/science.290.5497.1775 11099418

[B68] Chen FH Tuan RS Mesenchymal stem cells in arthritic diseases Arthritis Res Ther 2008 10 5 223 10.1186/ar2514 18947375 PMC2592798

[B69] Tan SC Pan WX Ma G Cai N Leong KW Liao K Viscoelastic behaviour of human mesenchymal stem cells BMC Cell Biol 2008 9 40 10.1186/1471-2121-9-40 18644160 PMC2500016

[B70] Boquest AC Noer A Collas P Epigenetic programming of mesenchymal stem cells from human adipose tissue Stem Cell Rev 2006 2 4 319 329 10.1007/BF02698059 17848719

[B71] Mizuno H Adipose-derived stem cells for tissue repair and regeneration: ten years of research and a literature review J Nippon Med Sch 2009 76 2 56 66 10.1272/jnms.76.56 19443990

[B72] Meirelles Lda S Nardi NB Methodology, biology and clinical applications of mesenchymal stem cells Front Biosci 2009 14 4281 4298 10.2741/3528 19273350

[B73] Ruhil S Kumar V Rathee P Umbilical cord stem cell: an overview Curr Pharm Biotechnol 2009 10 3 327 334 10.2174/138920109787847529 19355943

[B74] Mizoguchi M Ikeda S Suga Y Ogawa H Expression of cytokeratins and cornified cell envelope-associated proteins in umbilical cord epithelium: a comparative study of the umbilical cord, amniotic epithelia and fetal skin J Invest Dermatol 2000 115 1 133 134 10.1046/j.1523-1747.2000.00031-4.x 10886524

[B75] Hoyes AD Ultrastructure of the epithelium of the human umbilical cord J Anat 1969 105 Pt 1 149 162 5803186 PMC1232094

[B76] Mizoguchi M Suga Y Sanmano B Ikeda S Ogawa H Organotypic culture and surface plantation using umbilical cord epithelial cells: morphogenesis and expression of differentiation markers mimicking cutaneous epidermis J Dermatol Sci 2004 35 3 199 206 10.1016/j.jdermsci.2004.06.003 15381241

[B77] Sanmano B Mizoguchi M Suga Y Ikeda S Ogawa H Engraftment of umbilical cord epithelial cells in athymic mice: in an attempt to improve reconstructed skin equivalents used as epithelial composite J Dermatol Sci 2005 37 1 29 39 10.1016/j.jdermsci.2004.10.008 15619432

[B78] Ruetze M Gallinat S Lim IJ Chow E Phan TT Staeb F Wenck H Deppert W Knott A Common features of umbilical cord epithelial cells and epidermal keratinocytes J Dermatol Sci 2008 50 3 227 231 10.1016/j.jdermsci.2007.12.006 18242061

[B79] Mihu CM Mihu D Costin N Rus Ciuca D Susman S Ciortea R Isolation and characterization of stem cells from the placenta and the umbilical cord Rom J Morphol Embryol 2008 49 4 441 446 19050791

[B80] In 't Anker PS Scherjon SA Kleijburg-van der Keur C de Groot-Swings GM Claas FH Fibbe WE Kanhai HH Isolation of mesenchymal stem cells of fetal or maternal origin from human placenta Stem Cells 2004 22 7 1338 1345 15579651 10.1634/stemcells.2004-0058

[B81] Chien JW Duncan S Williams KM Pavletic SZ Bronchiolitis Obliterans Syndrome After Allogeneic Hematopoietic Stem Cell Transplantation - An Increasingly Recognized Manifestation of Chronic Graft-versus-Host Disease Biol Blood Marrow Transplant 2010 16 1 Suppl S106 14 10.1016/j.bbmt.2009.11.002 19896545 PMC3189470

[B82] Moghadam KG Marghoob B Alimoghadam K Shirani S Ghavamzadeh A Bronchiolitis obliterans following hematopoietic stem cell transplantation Hematol Oncol Stem Cell Ther 2010 3 2 100 101 20543546 10.1016/s1658-3876(10)50044-x

[B83] Miyagawa-Hayashino A Sonobe M Kubo T Yoshizawa A Date H Manabe T Non-specific interstitial pneumonia as a manifestation of graft-versus-host disease following pediatric allogeneic hematopoietic stem cell transplantation Pathol Int 2010 60 2 137 142 10.1111/j.1440-1827.2009.02492.x 20398200

[B84] Bryant DH Obliterative bronchiolitis in haematopoietic stem cell transplantation: can it be treated? Eur Respir J 2005 25 3 402 404 10.1183/09031936.05.00146604 15738280

[B85] Park M Koh KN Kim BE Im HJ Seo JJ Clinical features of late onset non-infectious pulmonary complications following pediatric allogeneic hematopoietic stem cell transplantation Clin Transplant 2010 10.1111/j.1399-0012.2010.01357.x 21077955

[B86] Yoshihara S Yanik G Cooke KR Mineishi S Bronchiolitis obliterans syndrome (BOS), bronchiolitis obliterans organizing pneumonia (BOOP), and other late-onset noninfectious pulmonary complications following allogeneic hematopoietic stem cell transplantation Biol Blood Marrow Transplant 2007 13 7 749 759 10.1016/j.bbmt.2007.05.001 17580252

[B87] Majeski EI Paintlia MK Lopez AD Harley RA London SD London L Respiratory reovirus 1/L induction of intraluminal fibrosis, a model of bronchiolitis obliterans organizing pneumonia, is dependent on T lymphocytes Am J Pathol 2003 163 4 1467 1479 14507654 10.1016/S0002-9440(10)63504-3 PMC1868312

[B88] Ditschkowski M Elmaagacli AH Trenschel R Peceny R Koldehoff M Schulte C Beelen DW T-cell depletion prevents from bronchiolitis obliterans and bronchiolitis obliterans with organizing pneumonia after allogeneic hematopoietic stem cell transplantation with related donors Haematologica 2007 92 4 558 561 10.3324/haematol.10710 17488669

[B89] Kanda Y Takahashi T Imai Y Miyagawa K Ohishi N Oka T Chiba S Hirai H Yazaki Y Bronchiolitis obliterans organizing pneumonia after syngeneic bone marrow transplantation for acute lymphoblastic leukemia Bone Marrow Transplant 1997 19 12 1251 1253 10.1038/sj.bmt.1700816 9208121

[B90] Cordier JF Bronchiolitis obliterans organizing pneumonia Semin Respir Crit Care Med 2000 21 2 135 146 10.1055/s-2000-9840 16088727

[B91] Patriarca F Skert C Bonifazi F Sperotto A Fili C Stanzani M Zaja F Cerno M Geromin A Bandini G Effect on survival of the development of late-onset non-infectious pulmonary complications after stem cell transplantation Haematologica 2006 91 9 1268 1272 16956831

[B92] Ferrara JL Levine JE Reddy P Holler E Graft-versus-host disease Lancet 2009 373 9674 1550 1561 10.1016/S0140-6736(09)60237-3 19282026 PMC2735047

[B93] Ferrara JL Deeg HJ Graft-versus-host disease N Engl J Med 1991 324 10 667 674 10.1056/NEJM199103073241005 1994250

[B94] Goker H Haznedaroglu IC Chao NJ Acute graft-vs-host disease: pathobiology and management Exp Hematol 2001 29 3 259 277 10.1016/S0301-472X(00)00677-9 11274753

[B95] Nevo S Enger C Swan V Wojno KJ Fuller AK Altomonte V Braine HG Noga SJ Vogelsang GB Acute bleeding after allogeneic bone marrow transplantation: association with graft versus host disease and effect on survival Transplantation 1999 67 5 681 689 10.1097/00007890-199903150-00007 10096522

[B96] Fujii N Takenaka K Shinagawa K Ikeda K Maeda Y Sunami K Hiramatsu Y Matsuo K Ishimaru F Niiya K Hepatic graft-versus-host disease presenting as an acute hepatitis after allogeneic peripheral blood stem cell transplantation Bone Marrow Transplant 2001 27 9 1007 1010 10.1038/sj.bmt.1702997 11436113

[B97] Lee JW Joachim Deeg H Prevention of chronic GVHD Best Pract Res Clin Haematol 2008 21 2 259 270 10.1016/j.beha.2008.02.010 18503991

[B98] Lee SJ New approaches for preventing and treating chronic graft-versus-host disease Blood 2005 105 11 4200 4206 10.1182/blood-2004-10-4023 15701727 PMC1895039

[B99] Martin PJ Weisdorf D Przepiorka D Hirschfeld S Farrell A Rizzo JD Foley R Socie G Carter S Couriel D National Institutes of Health Consensus Development Project on Criteria for Clinical Trials in Chronic Graft-versus-Host Disease: VI. Design of Clinical Trials Working Group report Biol Blood Marrow Transplant 2006 12 5 491 505 10.1016/j.bbmt.2006.03.004 16635784

[B100] Rimkus C Acute complications of stem cell transplant Semin Oncol Nurs 2009 25 2 129 138 10.1016/j.soncn.2009.03.007 19411016

[B101] Tabbara IA Zimmerman K Morgan C Nahleh Z Allogeneic hematopoietic stem cell transplantation: complications and results Arch Intern Med 2002 162 14 1558 1566 10.1001/archinte.162.14.1558 12123398

[B102] Skotnicki AB Krawczyk J Veno-occlusive disease--an important complication in hematopoietic cells transplantation Przegl Lek 2001 58 11 995 999 11987843

[B103] Lee SH Yoo KH Sung KW Koo HH Kwon YJ Kwon MM Park HJ Park BK Kim YY Park JA Hepatic veno-occlusive disease in children after hematopoietic stem cell transplantation: incidence, risk factors, and outcome Bone Marrow Transplant 2010 45 8 1287 1293 10.1038/bmt.2009.349 20010866

[B104] Cesaro S Pillon M Talenti E Toffolutti T Calore E Tridello G Strugo L Destro R Gazzola MV Varotto S A prospective survey on incidence, risk factors and therapy of hepatic veno-occlusive disease in children after hematopoietic stem cell transplantation Haematologica 2005 90 10 1396 1404 16219577

[B105] Shah MS Jeevangi NK Joshi A Khattry N Late-onset hepatic veno-occlusive disease post autologous peripheral stem cell transplantation successfully treated with oral defibrotide J Cancer Res Ther 2009 5 4 312 314 10.4103/0973-1482.59910 20160371

[B106] Lakshminarayanan S Sahdev I Goyal M Vlachos A Atlas M Lipton JM Low incidence of hepatic veno-occlusive disease in pediatric patients undergoing hematopoietic stem cell transplantation attributed to a combination of intravenous heparin, oral glutamine, and ursodiol at a single transplant institution Pediatr Transplant 2010 14 5 618 621 10.1111/j.1399-3046.2009.01285.x 20051023

[B107] Pittenger MF Martin BJ Mesenchymal stem cells and their potential as cardiac therapeutics Circ Res 2004 95 1 9 20 10.1161/01.RES.0000135902.99383.6f 15242981

[B108] Di Nicola M Carlo-Stella C Magni M Milanesi M Longoni PD Matteucci P Grisanti S Gianni AM Human bone marrow stromal cells suppress T-lymphocyte proliferation induced by cellular or nonspecific mitogenic stimuli Blood 2002 99 10 3838 3843 10.1182/blood.V99.10.3838 11986244

[B109] Alberio R Campbell KH Johnson AD Reprogramming somatic cells into stem cells Reproduction 2006 132 5 709 720 10.1530/rep.1.01077 17071772

[B110] Fairchild PJ Cartland S Nolan KF Waldmann H Embryonic stem cells and the challenge of transplantation tolerance Trends Immunol 2004 25 9 465 470 10.1016/j.it.2004.07.005 15324738

[B111] Amariglio N Hirshberg A Scheithauer BW Cohen Y Loewenthal R Trakhtenbrot L Paz N Koren-Michowitz M Waldman D Leider-Trejo L Donor-derived brain tumor following neural stem cell transplantation in an ataxia telangiectasia patient PLoS Med 2009 6 2 e1000029 10.1371/journal.pmed.1000029 19226183 PMC2642879

[B112] Lindvall O Kokaia Z Stem cells for the treatment of neurological disorders Nature 2006 441 7097 1094 1096 10.1038/nature04960 16810245

[B113] Lindvall O Kokaia Z Martinez-Serrano A Stem cell therapy for human neurodegenerative disorders-how to make it work Nat Med 2004 10 Suppl S42 50 10.1038/nm1064 15272269

[B114] Bjorklund LM Sanchez-Pernaute R Chung S Andersson T Chen IY McNaught KS Brownell AL Jenkins BG Wahlestedt C Kim KS Embryonic stem cells develop into functional dopaminergic neurons after transplantation in a Parkinson rat model Proc Natl Acad Sci USA 2002 99 4 2344 2349 10.1073/pnas.022438099 11782534 PMC122367

[B115] Arnhold S Lenartz D Kruttwig K Klinz FJ Kolossov E Hescheler J Sturm V Andressen C Addicks K Differentiation of green fluorescent protein-labeled embryonic stem cell-derived neural precursor cells into Thy-1-positive neurons and glia after transplantation into adult rat striatum J Neurosurg 2000 93 6 1026 1032 10.3171/jns.2000.93.6.1026 11117845

[B116] Werbowetski-Ogilvie TE Bosse M Stewart M Schnerch A Ramos-Mejia V Rouleau A Wynder T Smith MJ Dingwall S Carter T Characterization of human embryonic stem cells with features of neoplastic progression Nat Biotechnol 2009 27 1 91 97 10.1038/nbt.1516 19122652

[B117] Crooks VA Snyder J Regulating medical tourism Lancet 2010 376 9751 1465 1466 10.1016/S0140-6736(10)61993-9 21036273

[B118] Barclay E Stem-cell experts raise concerns about medical tourism Lancet 2009 373 9667 883 884 10.1016/S0140-6736(09)60529-8 19291840

[B119] Lau D Ogbogu U Taylor B Stafinski T Menon D Caulfield T Stem cell clinics online: the direct-to-consumer portrayal of stem cell medicine Cell Stem Cell 2008 3 6 591 594 10.1016/j.stem.2008.11.001 19041775

[B120] Pepper MS Cell-based therapy - navigating troubled waters S Afr Med J 2010 100 5 286 288 20460017 10.7196/samj.4131

[B121] Woo P Systemic juvenile idiopathic arthritis: diagnosis, management, and outcome Nat Clin Pract Rheumatol 2006 2 1 28 34 10.1038/ncprheum0084 16932649

[B122] Ringe J Sittinger M Tissue engineering in the rheumatic diseases Arthritis Res Ther 2009 11 1 211 10.1186/ar2572 19232063 PMC2688224

[B123] Hayward K Wallace CA Recent developments in anti-rheumatic drugs in pediatrics: treatment of juvenile idiopathic arthritis Arthritis Res Ther 2009 11 1 216 10.1186/ar2619 19291269 PMC2688259

[B124] Snowden JA Passweg J Moore JJ Milliken S Cannell P Van Laar J Verburg R Szer J Taylor K Joske D Autologous hemopoietic stem cell transplantation in severe rheumatoid arthritis: a report from the EBMT and ABMTR J Rheumatol 2004 31 3 482 488 14994391

[B125] Moore J Brooks P Milliken S Biggs J Ma D Handel M Cannell P Will R Rule S Joske D A pilot randomized trial comparing CD34-selected versus unmanipulated hemopoietic stem cell transplantation for severe, refractory rheumatoid arthritis Arthritis Rheum 2002 46 9 2301 2309 10.1002/art.10495 12355477

[B126] De Kleer IM Brinkman DM Ferster A Abinun M Quartier P Van Der Net J Ten Cate R Wedderburn LR Horneff G Oppermann J Autologous stem cell transplantation for refractory juvenile idiopathic arthritis: analysis of clinical effects, mortality, and transplant related morbidity Ann Rheum Dis 2004 63 10 1318 1326 10.1136/ard.2003.017798 15361393 PMC1754760

[B127] Jallouli M Frigui M Hmida MB Marzouk S Kaddour N Bahloul Z Clinical and immunological manifestations of systemic lupus erythematosus: study on 146 south Tunisian patients Saudi J Kidney Dis Transpl 2008 19 6 1001 1008 18974596

[B128] Ioannou Y Isenberg DA Current concepts for the management of systemic lupus erythematosus in adults: a therapeutic challenge Postgrad Med J 2002 78 924 599 606 10.1136/pmj.78.924.599 12415083 PMC1742538

[B129] Traynor AE Barr WG Rosa RM Rodriguez J Oyama Y Baker S Brush M Burt RK Hematopoietic stem cell transplantation for severe and refractory lupus. Analysis after five years and fifteen patients Arthritis Rheum 2002 46 11 2917 2923 10.1002/art.10594 12428232

[B130] Burt RK Traynor A Statkute L Barr WG Rosa R Schroeder J Verda L Krosnjar N Quigley K Yaung K Nonmyeloablative hematopoietic stem cell transplantation for systemic lupus erythematosus JAMA 2006 295 5 527 535 10.1001/jama.295.5.527 16449618

[B131] Goldblatt F Isenberg DA New therapies for systemic lupus erythematosus Clin Exp Immunol 2005 140 2 205 212 10.1111/j.1365-2249.2005.02795.x 15807843 PMC1809371

[B132] Compston A Coles A Multiple sclerosis Lancet 2008 372 9648 1502 1517 10.1016/S0140-6736(08)61620-7 18970977

[B133] Katsara M Matsoukas J Deraos G Apostolopoulos V Towards immunotherapeutic drugs and vaccines against multiple sclerosis Acta Biochim Biophys Sin (Shanghai) 2008 40 7 636 642 10.1111/j.1745-7270.2008.00444.x 18604455

[B134] Ebers GC Natural history of primary progressive multiple sclerosis Mult Scler 2004 10 Suppl 1 S8 13 discussion S13-15 10.1177/135245850401000103 15218804

[B135] Saccardi R Mancardi GL Solari A Bosi A Bruzzi P Di Bartolomeo P Donelli A Filippi M Guerrasio A Gualandi F Autologous HSCT for severe progressive multiple sclerosis in a multicenter trial: impact on disease activity and quality of life Blood 2005 105 6 2601 2607 10.1182/blood-2004-08-3205 15546956

[B136] Fassas A Passweg JR Anagnostopoulos A Kazis A Kozak T Havrdova E Carreras E Graus F Kashyap A Openshaw H Hematopoietic stem cell transplantation for multiple sclerosis. A retrospective multicenter study J Neurol 2002 249 8 1088 1097 10.1007/s00415-002-0800-7 12195460

[B137] Fassas A Anagnostopoulos A Kazis A Kapinas K Sakellari I Kimiskidis V Smias C Eleftheriadis N Tsimourtou V Autologous stem cell transplantation in progressive multiple sclerosis--an interim analysis of efficacy J Clin Immunol 2000 20 1 24 30 10.1023/A:1006686426090 10798604

[B138] Mezey E Chandross KJ Harta G Maki RA McKercher SR Turning blood into brain: cells bearing neuronal antigens generated in vivo from bone marrow Science 2000 290 5497 1779 1782 10.1126/science.290.5497.1779 11099419

[B139] Lim IG Schrieber L Management of systemic sclerosis Isr Med Assoc J 2002 4 11 Suppl 953 957 12455189

[B140] Akerkar SM Bichile LS Therapeutic options for systemic sclerosis Indian J Dermatol Venereol Leprol 2004 70 2 67 75 17642568

[B141] Tyndall A Black C Finke J Winkler J Mertlesmann R Peter HH Gratwohl A Treatment of systemic sclerosis with autologous haemopoietic stem cell transplantation Lancet 1997 349 9047 254 10.1016/S0140-6736(05)64864-7 9014919

[B142] van den Hoogen FH van de Putte LB Treatment of systemic sclerosis Curr Opin Rheumatol 1994 6 6 637 641 10.1097/00002281-199411000-00015 7865386

[B143] Martini A Maccario R Ravelli A Montagna D De Benedetti F Bonetti F Viola S Zecca M Perotti C Locatelli F Marked and sustained improvement two years after autologous stem cell transplantation in a girl with systemic sclerosis Arthritis Rheum 1999 42 4 807 811 10.1002/1529-0131(199904)42:4<807::AID-ANR26>3.0.CO;2-T 10211898

[B144] Binks M Passweg JR Furst D McSweeney P Sullivan K Besenthal C Finke J Peter HH van Laar J Breedveld FC Phase I/II trial of autologous stem cell transplantation in systemic sclerosis: procedure related mortality and impact on skin disease Ann Rheum Dis 2001 60 6 577 584 10.1136/ard.60.6.577 11350846 PMC1753658

[B145] Farge D Marolleau JP Zohar S Marjanovic Z Cabane J Mounier N Hachulla E Philippe P Sibilia J Rabian C Autologous bone marrow transplantation in the treatment of refractory systemic sclerosis: early results from a French multicentre phase I-II study Br J Haematol 2002 119 3 726 739 10.1046/j.1365-2141.2002.03895.x 12437652

[B146] McSweeney PA Nash RA Sullivan KM Storek J Crofford LJ Dansey R Mayes MD McDonagh KT Nelson JL Gooley TA High-dose immunosuppressive therapy for severe systemic sclerosis: initial outcomes Blood 2002 100 5 1602 1610 12176878 PMC2956742

[B147] Farge D Passweg J van Laar JM Marjanovic Z Besenthal C Finke J Peter HH Breedveld FC Fibbe WE Black C Autologous stem cell transplantation in the treatment of systemic sclerosis: report from the EBMT/EULAR Registry Ann Rheum Dis 2004 63 8 974 981 10.1136/ard.2003.011205 15249325 PMC1755096

[B148] Rampton DS Management of Crohn's disease BMJ 1999 319 7223 1480 1485 10582935 10.1136/bmj.319.7223.1480 PMC1117206

[B149] Cassinotti A Annaloro C Ardizzone S Onida F Della Volpe A Clerici M Usardi P Greco S Maconi G Porro GB Autologous haematopoietic stem cell transplantation without CD34+ cell selection in refractory Crohn's disease Gut 2008 57 2 211 217 10.1136/gut.2007.128694 17895357

[B150] Oyama Y Craig RM Traynor AE Quigley K Statkute L Halverson A Brush M Verda L Kowalska B Krosnjar N Autologous hematopoietic stem cell transplantation in patients with refractory Crohn's disease Gastroenterology 2005 128 3 552 563 10.1053/j.gastro.2004.11.051 15765390

[B151] Burt RK Traynor A Oyama Y Craig R High-dose immune suppression and autologous hematopoietic stem cell transplantation in refractory Crohn disease Blood 2003 101 5 2064 2066 10.1182/blood-2002-07-2122 12393477

[B152] Stasi R Provan D Management of immune thrombocytopenic purpura in adults Mayo Clin Proc 2004 79 4 504 522 10.4065/79.4.504 15065616

[B153] Huhn RD Fogarty PF Nakamura R Read EJ Leitman SF Rick ME Kimball J Greene A Hansmann K Gratwohl A High-dose cyclophosphamide with autologous lymphocyte-depleted peripheral blood stem cell (PBSC) support for treatment of refractory chronic autoimmune thrombocytopenia Blood 2003 101 1 71 77 10.1182/blood-2001-12-0171 12393623

[B154] Urban C Lackner H Sovinz P Benesch M Schwinger W Dornbusch HJ Moser A Successful unrelated cord blood transplantation in a 7-year-old boy with Evans syndrome refractory to immunosuppression and double autologous stem cell transplantation Eur J Haematol 2006 76 6 526 530 10.1111/j.0902-4441.2006.t01-1-EJH2549.x 16529601

[B155] Riechsteiner G Speich R Schanz U Russi EW Weder W Boehler A Haemolytic anaemia after lung transplantation: an immune-mediated phenomenon? Swiss Med Wkly 2003 133 9-10 143 147 12707841 10.4414/smw.2003.10123

[B156] Pratt G Kinsey SE Remission of severe, intractable autoimmune haemolytic anaemia following matched unrelated donor transplantation Bone Marrow Transplant 2001 28 8 791 793 10.1038/sj.bmt.1703232 11781633

[B157] Sallah S Wan JY Hanrahan LR Future development of lymphoproliferative disorders in patients with autoimmune hemolytic anemia Clin Cancer Res 2001 7 4 791 794 11309323

[B158] Seeliger S Baumann M Mohr M Jurgens H Frosch M Vormoor J Autologous peripheral blood stem cell transplantation and anti-B-cell directed immunotherapy for refractory auto-immune haemolytic anaemia Eur J Pediatr 2001 160 8 492 496 10.1007/s004310100778 11548187

[B159] Passweg JR Rabusin M Musso M Beguin Y Cesaro S Ehninger G Espigado I Iriondo A Jost L Koza V Haematopoetic stem cell transplantation for refractory autoimmune cytopenia Br J Haematol 2004 125 6 749 755 10.1111/j.1365-2141.2004.04978.x 15180864

[B160] Eisenbarth GS Update in type 1 diabetes J Clin Endocrinol Metab 2007 92 7 2403 2407 10.1210/jc.2007-0339 17616634

[B161] Aiello LP Gardner TW King GL Blankenship G Cavallerano JD Ferris FL Klein R Diabetic retinopathy Diabetes Care 1998 21 1 143 156 9538986 10.2337/diacare.21.1.143

[B162] Sima AA Zhang W Grunberger G Type 1 diabetic neuropathy and C-peptide Exp Diabesity Res 2004 5 1 65 77 10.1080/15438600490424541 15198372 PMC2478622

[B163] Ingberg CM Palmer M Schvarcz E Aman J Prevalence of urinary tract symptoms in long-standing type 1 diabetes mellitus Diabetes Metab 1998 24 4 351 354 9805646

[B164] Couri CE Oliveira MC Stracieri AB Moraes DA Pieroni F Barros GM Madeira MI Malmegrim KC Foss-Freitas MC Simoes BP C-peptide levels and insulin independence following autologous nonmyeloablative hematopoietic stem cell transplantation in newly diagnosed type 1 diabetes mellitus JAMA 2009 301 15 1573 1579 10.1001/jama.2009.470 19366777

[B165] Snarski E Torosian T Paluszewska M Urbanowska E Milczarczyk A Jedynasty K Franek E Jedrzejczak WW Alleviation of exogenous insulin requirement in type 1 diabetes mellitus after immunoablation and transplantation of autologous hematopoietic stem cells Pol Arch Med Wewn 2009 119 6 422 426 19694226

[B166] Trivedi HL Vanikar AV Thakker U Firoze A Dave SD Patel CN Patel JV Bhargava AB Shankar V Human adipose tissue-derived mesenchymal stem cells combined with hematopoietic stem cell transplantation synthesize insulin Transplant Proc 2008 40 4 1135 1139 10.1016/j.transproceed.2008.03.113 18555133

[B167] Wijesekera LC Leigh PN Amyotrophic lateral sclerosis Orphanet J Rare Dis 2009 4 3 10.1186/1750-1172-4-3 19192301 PMC2656493

[B168] Janson CG Ramesh TM During MJ Leone P Heywood J Human intrathecal transplantation of peripheral blood stem cells in amyotrophic lateral sclerosis J Hematother Stem Cell Res 2001 10 6 913 915 10.1089/152581601317211015 11798518

[B169] Mazzini L Ferrero I Luparello V Rustichelli D Gunetti M Mareschi K Testa L Stecco A Tarletti R Miglioretti M Mesenchymal stem cell transplantation in amyotrophic lateral sclerosis: A Phase I clinical trial Exp Neurol 2010 223 1 229 37 10.1016/j.expneurol.2009.08.007 19682989

[B170] Mazzini L Fagioli F Boccaletti R Mareschi K Oliveri G Olivieri C Pastore I Marasso R Madon E Stem cell therapy in amyotrophic lateral sclerosis: a methodological approach in humans Amyotroph Lateral Scler Other Motor Neuron Disord 2003 4 3 158 161 10.1080/14660820310014653 13129802

[B171] Martinez HR Gonzalez-Garza MT Moreno-Cuevas JE Caro E Gutierrez-Jimenez E Segura JJ Stem-cell transplantation into the frontal motor cortex in amyotrophic lateral sclerosis patients Cytotherapy 2009 11 1 26 34 10.1080/14653240802644651 19191058

[B172] Papadeas ST Maragakis NJ Advances in stem cell research for Amyotrophic Lateral Sclerosis Curr Opin Biotechnol 2009 20 5 545 551 10.1016/j.copbio.2009.09.003 19819686

[B173] Astradsson A Cooper O Vinuela A Isacson O Recent advances in cell-based therapy for Parkinson disease Neurosurg Focus 2008 24 3-4 E6 10.3171/FOC/2008/24/3-4/E5 18341409

[B174] Weintraub D Comella CL Horn S Parkinson's disease--Part 2: Treatment of motor symptoms Am J Manag Care 2008 14 2 Suppl S49 58 18402508

[B175] Hagell P Schrag A Piccini P Jahanshahi M Brown R Rehncrona S Widner H Brundin P Rothwell JC Odin P Sequential bilateral transplantation in Parkinson's disease: effects of the second graft Brain 1999 122 Pt 6 1121 1132 10.1093/brain/122.6.1121 10356064

[B176] Brundin P Pogarell O Hagell P Piccini P Widner H Schrag A Kupsch A Crabb L Odin P Gustavii B Bilateral caudate and putamen grafts of embryonic mesencephalic tissue treated with lazaroids in Parkinson's disease Brain 2000 123 Pt 7 1380 1390 10.1093/brain/123.7.1380 10869050

[B177] Freed CR Greene PE Breeze RE Tsai WY DuMouchel W Kao R Dillon S Winfield H Culver S Trojanowski JQ Transplantation of embryonic dopamine neurons for severe Parkinson's disease N Engl J Med 2001 344 10 710 719 10.1056/NEJM200103083441002 11236774

[B178] Hauser RA Freeman TB Snow BJ Nauert M Gauger L Kordower JH Olanow CW Long-term evaluation of bilateral fetal nigral transplantation in Parkinson disease Arch Neurol 1999 56 2 179 187 10.1001/archneur.56.2.179 10025423

[B179] Olanow CW Goetz CG Kordower JH Stoessl AJ Sossi V Brin MF Shannon KM Nauert GM Perl DP Godbold J A double-blind controlled trial of bilateral fetal nigral transplantation in Parkinson's disease Ann Neurol 2003 54 3 403 414 10.1002/ana.10720 12953276

[B180] Lima C Pratas-Vital J Escada P Hasse-Ferreira A Capucho C Peduzzi JD Olfactory mucosa autografts in human spinal cord injury: a pilot clinical study J Spinal Cord Med 2006 29 3 191 203 discussion 204-196 16859223 10.1080/10790268.2006.11753874 PMC1864811

[B181] Mackay-Sim A Feron F Cochrane J Bassingthwaighte L Bayliss C Davies W Fronek P Gray C Kerr G Licina P Autologous olfactory ensheathing cell transplantation in human paraplegia: a 3-year clinical trial Brain 2008 131 Pt 9 2376 2386 10.1093/brain/awn173 18689435 PMC2525447

[B182] Yoon SH Shim YS Park YH Chung JK Nam JH Kim MO Park HC Park SR Min BH Kim EY Complete spinal cord injury treatment using autologous bone marrow cell transplantation and bone marrow stimulation with granulocyte macrophage-colony stimulating factor: Phase I/II clinical trial Stem Cells 2007 25 8 2066 2073 10.1634/stemcells.2006-0807 17464087

[B183] Freeman TB Cicchetti F Hauser RA Deacon TW Li XJ Hersch SM Nauert GM Sanberg PR Kordower JH Saporta S Transplanted fetal striatum in Huntington's disease: phenotypic development and lack of pathology Proc Natl Acad Sci USA 2000 97 25 13877 13882 10.1073/pnas.97.25.13877 11106399 PMC17669

[B184] Kopyov OV Jacques S Lieberman A Duma CM Eagle KS Safety of intrastriatal neurotransplantation for Huntington's disease patients Exp Neurol 1998 149 1 97 108 10.1006/exnr.1997.6685 9454619

[B185] Rosser AE Barker RA Harrower T Watts C Farrington M Ho AK Burnstein RM Menon DK Gillard JH Pickard J Unilateral transplantation of human primary fetal tissue in four patients with Huntington's disease: NEST-UK safety report ISRCTN no 36485475 J Neurol Neurosurg Psychiatry 2002 73 6 678 685 10.1136/jnnp.73.6.678 12438470 PMC1757375

[B186] Gaura V Bachoud-Levi AC Ribeiro MJ Nguyen JP Frouin V Baudic S Brugieres P Mangin JF Boisse MF Palfi S Striatal neural grafting improves cortical metabolism in Huntington's disease patients Brain 2004 127 Pt 1 65 72 10.1093/brain/awh003 14607797

[B187] Palfi S Nguyen JP Brugieres P Le Guerinel C Hantraye P Remy P Rostaing S Defer GL Cesaro P Keravel Y MRI-stereotactical approach for neural grafting in basal ganglia disorders Exp Neurol 1998 150 2 272 281 10.1006/exnr.1997.6754 9527897

[B188] Hauser RA Sandberg PR Freeman TB Stoessl AJ Bilateral human fetal striatal transplantation in Huntington's disease Neurology 2002 58 11 1704 author reply 1704 12058114 10.1212/wnl.58.11.1704

[B189] Rabinovich SS Seledtsov VI Banul NV Poveshchenko OV Senyukov VV Astrakov SV Samarin DM Taraban VY Cell therapy of brain stroke Bull Exp Biol Med 2005 139 1 126 128 10.1007/s10517-005-0229-y 16142294

[B190] Bang OY Lee JS Lee PH Lee G Autologous mesenchymal stem cell transplantation in stroke patients Ann Neurol 2005 57 6 874 882 10.1002/ana.20501 15929052

[B191] Shyu WC Lin SZ Lee CC Liu DD Li H Granulocyte colony-stimulating factor for acute ischemic stroke: a randomized controlled trial CMAJ 2006 174 7 927 933 16517764 10.1503/cmaj.051322 PMC1405861

[B192] Yiu EM Kornberg AJ Duchenne muscular dystrophy Neurol India 2008 56 3 236 247 10.4103/0028-3886.43441 18974549

[B193] Torrente Y Belicchi M Marchesi C Dantona G Cogiamanian F Pisati F Gavina M Giordano R Tonlorenzi R Fagiolari G Autologous transplantation of muscle-derived CD133+ stem cells in Duchenne muscle patients Cell Transplant 2007 16 6 563 577 17912948 10.3727/000000007783465064

[B194] Neumeyer AM Cros D McKenna-Yasek D Zawadzka A Hoffman EP Pegoraro E Hunter RG Munsat TL Brown RH Jr Pilot study of myoblast transfer in the treatment of Becker muscular dystrophy Neurology 1998 51 2 589 592 9710042 10.1212/wnl.51.2.589

[B195] Gussoni E Blau HM Kunkel LM The fate of individual myoblasts after transplantation into muscles of DMD patients Nat Med 1997 3 9 970 977 10.1038/nm0997-970 9288722

[B196] Miller RG Sharma KR Pavlath GK Gussoni E Mynhier M Lanctot AM Greco CM Steinman L Blau HM Myoblast implantation in Duchenne muscular dystrophy: the San Francisco study Muscle Nerve 1997 20 4 469 478 10.1002/(SICI)1097-4598(199704)20:4<469::AID-MUS10>3.0.CO;2-U 9121505

[B197] Mendell JR Kissel JT Amato AA King W Signore L Prior TW Sahenk Z Benson S McAndrew PE Rice R Myoblast transfer in the treatment of Duchenne's muscular dystrophy N Engl J Med 1995 333 13 832 838 10.1056/NEJM199509283331303 7651473

[B198] Tremblay JP Malouin F Roy R Huard J Bouchard JP Satoh A Richards CL Results of a triple blind clinical study of myoblast transplantations without immunosuppressive treatment in young boys with Duchenne muscular dystrophy Cell Transplant 1993 2 2 99 112 8143083 10.1177/096368979300200203

[B199] Vincent R Advances in the early diagnosis and management of acute myocardial infarction J Accid Emerg Med 1996 13 2 74 79 8653254 10.1136/emj.13.2.74 PMC1342640

[B200] Goldman LE Eisenberg MJ Identification and management of patients with failed thrombolysis after acute myocardial infarction Ann Intern Med 2000 132 7 556 565 10744593 10.7326/0003-4819-132-7-200004040-00008

[B201] Menasche P Alfieri O Janssens S McKenna W Reichenspurner H Trinquart L Vilquin JT Marolleau JP Seymour B Larghero J The Myoblast Autologous Grafting in Ischemic Cardiomyopathy (MAGIC) trial: first randomized placebo-controlled study of myoblast transplantation Circulation 2008 117 9 1189 1200 10.1161/CIRCULATIONAHA.107.734103 18285565

[B202] Hagege AA Marolleau JP Vilquin JT Alheritiere A Peyrard S Duboc D Abergel E Messas E Mousseaux E Schwartz K Skeletal myoblast transplantation in ischemic heart failure: long-term follow-up of the first phase I cohort of patients Circulation 2006 114 1 Suppl I108 113 16820558 10.1161/CIRCULATIONAHA.105.000521

[B203] Siminiak T Kalawski R Fiszer D Jerzykowska O Rzezniczak J Rozwadowska N Kurpisz M Autologous skeletal myoblast transplantation for the treatment of postinfarction myocardial injury: phase I clinical study with 12 months of follow-up Am Heart J 2004 148 3 531 537 10.1016/j.ahj.2004.03.043 15389244

[B204] Schachinger V Assmus B Erbs S Elsasser A Haberbosch W Hambrecht R Yu J Corti R Mathey DG Hamm CW Intracoronary infusion of bone marrow-derived mononuclear cells abrogates adverse left ventricular remodelling post-acute myocardial infarction: insights from the reinfusion of enriched progenitor cells and infarct remodelling in acute myocardial infarction (REPAIR-AMI) trial Eur J Heart Fail 2009 11 10 973 979 10.1093/eurjhf/hfp113 19789401

[B205] Schachinger V Erbs S Elsasser A Haberbosch W Hambrecht R Holschermann H Yu J Corti R Mathey DG Hamm CW Intracoronary bone marrow-derived progenitor cells in acute myocardial infarction N Engl J Med 2006 355 12 1210 1221 10.1056/NEJMoa060186 16990384

[B206] Wollert KC Meyer GP Lotz J Ringes-Lichtenberg S Lippolt P Breidenbach C Fichtner S Korte T Hornig B Messinger D Intracoronary autologous bone-marrow cell transfer after myocardial infarction: the BOOST randomised controlled clinical trial Lancet 2004 364 9429 141 148 10.1016/S0140-6736(04)16626-9 15246726

[B207] Ang LP Tan DT Ocular surface stem cells and disease: current concepts and clinical applications Ann Acad Med Singapore 2004 33 5 576 580 15531952

[B208] Rama P Bonini S Lambiase A Golisano O Paterna P De Luca M Pellegrini G Autologous fibrin-cultured limbal stem cells permanently restore the corneal surface of patients with total limbal stem cell deficiency Transplantation 2001 72 9 1478 1485 10.1097/00007890-200111150-00002 11707733

[B209] Daya SM Ilari FA Living related conjunctival limbal allograft for the treatment of stem cell deficiency Ophthalmology 2001 108 1 126 133 discussion 133-124 10.1016/S0161-6420(00)00475-9 11150276

[B210] Ilari L Daya SM Long-term outcomes of keratolimbal allograft for the treatment of severe ocular surface disorders Ophthalmology 2002 109 7 1278 1284 10.1016/S0161-6420(02)01081-3 12093650

[B211] Solomon A Ellies P Anderson DF Touhami A Grueterich M Espana EM Ti SE Goto E Feuer WJ Tseng SC Long-term outcome of keratolimbal allograft with or without penetrating keratoplasty for total limbal stem cell deficiency Ophthalmology 2002 109 6 1159 1166 10.1016/S0161-6420(02)00960-0 12045060

[B212] Shimazaki J Maruyama F Shimmura S Fujishima H Tsubota K Immunologic rejection of the central graft after limbal allograft transplantation combined with penetrating keratoplasty Cornea 2001 20 2 149 152 10.1097/00003226-200103000-00006 11248817

[B213] Mobasheri A Csaki C Clutterbuck AL Rahmanzadeh M Shakibaei M Mesenchymal stem cells in connective tissue engineering and regenerative medicine: applications in cartilage repair and osteoarthritis therapy Histol Histopathol 2009 24 3 347 366 19130405 10.14670/HH-24.347

[B214] McNair PJ Simmonds MA Boocock MG Larmer PJ Exercise therapy for the management of osteoarthritis of the hip joint: a systematic review Arthritis Res Ther 2009 11 3 R98 10.1186/ar2743 19555502 PMC2714154

[B215] Srbely JZ Ultrasound in the management of osteoarthritis: part I: a review of the current literature J Can Chiropr Assoc 2008 52 1 30 37 18327300 PMC2258240

[B216] Barron MC Rubin BR Managing osteoarthritic knee pain J Am Osteopath Assoc 2007 107 10 Suppl 6 ES21 27 17986674

[B217] Santaguida PL Hawker GA Hudak PL Glazier R Mahomed NN Kreder HJ Coyte PC Wright JG Patient characteristics affecting the prognosis of total hip and knee joint arthroplasty: a systematic review Can J Surg 2008 51 6 428 436 19057730 PMC2592576

[B218] Centeno CJ Busse D Kisiday J Keohan C Freeman M Karli D Increased knee cartilage volume in degenerative joint disease using percutaneously implanted, autologous mesenchymal stem cells Pain Physician 2008 11 3 343 353 18523506

[B219] Schuppan D Afdhal NH Liver cirrhosis Lancet 2008 371 9615 838 851 10.1016/S0140-6736(08)60383-9 18328931 PMC2271178

[B220] Pai M Zacharoulis D Milicevic MN Helmy S Jiao LR Levicar N Tait P Scott M Marley SB Jestice K Autologous infusion of expanded mobilized adult bone marrow-derived CD34+ cells into patients with alcoholic liver cirrhosis Am J Gastroenterol 2008 103 8 1952 1958 10.1111/j.1572-0241.2008.01993.x 18637092

[B221] Lyra AC Soares MB da Silva LF Fortes MF Silva AG Mota AC Oliveira SA Braga EL de Carvalho WA Genser B Feasibility and safety of autologous bone marrow mononuclear cell transplantation in patients with advanced chronic liver disease World J Gastroenterol 2007 13 7 1067 1073 17373741 10.3748/wjg.v13.i7.1067 PMC4146869

[B222] am Esch JS Knoefel WT Klein M Ghodsizad A Fuerst G Poll LW Piechaczek C Burchardt ER Feifel N Stoldt V Portal application of autologous CD133+ bone marrow cells to the liver: a novel concept to support hepatic regeneration Stem Cells 2005 23 4 463 470 10.1634/stemcells.2004-0283 15790766

[B223] Terai S Ishikawa T Omori K Aoyama K Marumoto Y Urata Y Yokoyama Y Uchida K Yamasaki T Fujii Y Improved liver function in patients with liver cirrhosis after autologous bone marrow cell infusion therapy Stem Cells 2006 24 10 2292 2298 10.1634/stemcells.2005-0542 16778155

[B224] Heldwein FL McCullough TC Souto CA Galiano M Barret E Localized renal cell carcinoma management: an update Int Braz J Urol 2008 34 6 676 689 discussion 689-690 10.1590/S1677-55382008000600002 19111072

[B225] Oudard S George D Medioni J Motzer R Treatment options in renal cell carcinoma: past, present and future Ann Oncol 2007 18 Suppl 10 x25 31 10.1093/annonc/mdm411 17761720

[B226] Peccatori J Barkholt L Demirer T Sormani MP Bruzzi P Ciceri F Zambelli A Da Prada GA Pedrazzoli P Siena S Prognostic factors for survival in patients with advanced renal cell carcinoma undergoing nonmyeloablative allogeneic stem cell transplantation Cancer 2005 104 10 2099 2103 10.1002/cncr.21477 16220555

[B227] Barkholt L Bregni M Remberger M Blaise D Peccatori J Massenkeil G Pedrazzoli P Zambelli A Bay JO Francois S Allogeneic haematopoietic stem cell transplantation for metastatic renal carcinoma in Europe Ann Oncol 2006 17 7 1134 1140 10.1093/annonc/mdl086 16648196

[B228] Artz AS Van Besien K Zimmerman T Gajewski TF Rini BI Hu HS Stadler WM Vogelzang NJ Long-term follow-up of nonmyeloablative allogeneic stem cell transplantation for renal cell carcinoma: The University of Chicago Experience Bone Marrow Transplant 2005 35 3 253 260 10.1038/sj.bmt.1704760 15543195

[B229] Childs R Chernoff A Contentin N Bahceci E Schrump D Leitman S Read EJ Tisdale J Dunbar C Linehan WM Regression of metastatic renal-cell carcinoma after nonmyeloablative allogeneic peripheral-blood stem-cell transplantation N Engl J Med 2000 343 11 750 758 10.1056/NEJM200009143431101 10984562

[B230] Singletary SE Breast cancer management: the road to today Cancer 2008 113 7 Suppl 1844 1849 10.1002/cncr.23649 18798545

[B231] Biron P Durand M Roche H Delozier T Battista C Fargeot P Spaeth D Bachelot T Poiget E Monnot F Pegase 03: a prospective randomized phase III trial of FEC with or without high-dose thiotepa, cyclophosphamide and autologous stem cell transplantation in first-line treatment of metastatic breast cancer Bone Marrow Transplant 2008 41 6 555 562 10.1038/sj.bmt.1705935 18037940

[B232] Ueno NT Rizzo JD Demirer T Cheng YC Hegenbart U Zhang MJ Bregni M Carella A Blaise D Bashey A Allogeneic hematopoietic cell transplantation for metastatic breast cancer Bone Marrow Transplant 2008 41 6 537 545 10.1038/sj.bmt.1705940 18084340

[B233] Carella AM Bregni M Current role of allogeneic stem cell transplantation in breast cancer Ann Oncol 2007 18 10 1591 1593 10.1093/annonc/mdm342 17846023

[B234] Gill S Blackstock AW Goldberg RM Colorectal cancer Mayo Clin Proc 2007 82 1 114 129 10.4065/82.1.114 17285793

[B235] Benson AB Epidemiology, disease progression, and economic burden of colorectal cancer J Manag Care Pharm 2007 13 6 Suppl C S5 18 17713990 10.18553/jmcp.2007.13.s6-c.5 PMC10438323

[B236] Nagy VM Updating the management of rectal cancer J Gastrointestin Liver Dis 2008 17 1 69 74 18392247 10.1007/s11749-006-0023-9

[B237] Kojima R Kami M Hori A Murashige N Ohnishi M Kim SW Hamaki T Kishi Y Tsutsumi Y Masauzi N Reduced-intensity allogeneic hematopoietic stem-cell transplantation as an immunotherapy for metastatic colorectal cancer Transplantation 2004 78 12 1740 1746 10.1097/01.TP.0000146194.36297.4E 15614146

[B238] Aglietta M Barkholt L Schianca FC Caravelli D Omazic B Minotto C Leone F Hentschke P Bertoldero G Capaldi A Reduced-intensity allogeneic hematopoietic stem cell transplantation in metastatic colorectal cancer as a novel adoptive cell therapy approach. The European group for blood and marrow transplantation experience Biol Blood Marrow Transplant 2009 15 3 326 335 10.1016/j.bbmt.2008.11.036 19203723

[B239] Hashino S Kobayashi S Takahata M Onozawa M Nakagawa M Kawamura T Fujisawa F Izumiyama K Kahata K Kondo T Graft-versus-tumor effect after reduced-intensity allogeneic hematopoietic stem cell transplantation in a patient with advanced colon cancer Int J Clin Oncol 2008 13 2 176 180 10.1007/s10147-007-0716-4 18463966

[B240] Carnevale-Schianca F Cignetti A Capaldi A Vitaggio K Vallario A Ricchiardi A Sperti E Ferraris R Gatti M Grignani G Allogeneic nonmyeloablative hematopoietic cell transplantation in metastatic colon cancer: tumor-specific T cells directed to a tumor-associated antigen are generated in vivo during GVHD Blood 2006 107 9 3795 3803 10.1182/blood-2005-10-3945 16403911

[B241] Schilder RJ Boente MP Corn BW Lanciano RM Young RC Ozols RF The management of early ovarian cancer Oncology (Williston Park) 1995 9 2 171 182 discussion 185-177 8771099

[B242] Bay JO Fleury J Choufi B Tournilhac O Vincent C Bailly C Dauplat J Viens P Faucher C Blaise D Allogeneic hematopoietic stem cell transplantation in ovarian carcinoma: results of five patients Bone Marrow Transplant 2002 30 2 95 102 10.1038/sj.bmt.1703609 12132048

[B243] Rini BI Zimmerman T Stadler WM Gajewski TF Vogelzang NJ Allogeneic stem-cell transplantation of renal cell cancer after nonmyeloablative chemotherapy: feasibility, engraftment, and clinical results J Clin Oncol 2002 20 8 2017 2024 10.1200/JCO.2002.08.068 11956260

[B244] Papadimitriou C Dafni U Anagnostopoulos A Vlachos G Voulgaris Z Rodolakis A Aravantinos G Bamias A Bozas G Kiosses E High-dose melphalan and autologous stem cell transplantation as consolidation treatment in patients with chemosensitive ovarian cancer: results of a single-institution randomized trial Bone Marrow Transplant 2008 41 6 547 554 10.1038/sj.bmt.1705925 18026149

[B245] Sarosy GA Reed E Autologous stem-cell transplantation in ovarian cancer: is more better? Ann Intern Med 2000 133 7 555 556 11015170 10.7326/0003-4819-133-7-200010030-00016

[B246] Seidenfeld J Samson DJ Bonnell CJ Ziegler KM Aronson N Management of small cell lung cancer Evid Rep Technol Assess (Full Rep) 2006 143 1 154 17764204 PMC4781398

[B247] Souhami RL Hajichristou HT Miles DW Earl HM Harper PG Ash CM Goldstone AH Spiro SG Geddes DM Tobias JS Intensive chemotherapy with autologous bone marrow transplantation for small-cell lung cancer Cancer Chemother Pharmacol 1989 24 5 321 325 10.1007/BF00304766 2547528

[B248] Humblet Y Symann M Bosly A Delaunois L Francis C Machiels J Beauduin M Doyen C Weynants P Longueville J Late intensification chemotherapy with autologous bone marrow transplantation in selected small-cell carcinoma of the lung: a randomized study J Clin Oncol 1987 5 12 1864 1873 2824708 10.1200/JCO.1987.5.12.1864

[B249] Leyvraz S Perey L Rosti G Lange A Pampallona S Peters R Humblet Y Bosquee L Pasini F Marangolo M Multiple courses of high-dose ifosfamide, carboplatin, and etoposide with peripheral-blood progenitor cells and filgrastim for small-cell lung cancer: A feasibility study by the European Group for Blood and Marrow Transplantation J Clin Oncol 1999 17 11 3531 3539 10550151 10.1200/JCO.1999.17.11.3531

[B250] Brugger W Fetscher S Hasse J Frommhold H Pressler K Mertelsmann R Engelhardt R Kanz L Multimodality treatment including early high-dose chemotherapy with peripheral blood stem cell transplantation in limited-disease small cell lung cancer Semin Oncol 1998 25 1 Suppl 2 42 48 9535211

[B251] Hoelzer D Gokbuget N Ottmann O Pui CH Relling MV Appelbaum FR van Dongen JJ Szczepanski T Acute lymphoblastic leukemia Hematology Am Soc Hematol Educ Program 2002 162 192 12446423 10.1182/asheducation-2002.1.162

[B252] Stone RM O'Donnell MR Sekeres MA Acute myeloid leukemia Hematology Am Soc Hematol Educ Program 2004 98 117 15561679 10.1182/asheducation-2004.1.98

[B253] Shah NP Medical management of CML Hematology Am Soc Hematol Educ Program 2007 371 375 18024653 10.1182/asheducation-2007.1.371

[B254] Quintas-Cardama A Cortes JE Chronic myeloid leukemia: diagnosis and treatment Mayo Clin Proc 2006 81 7 973 988 10.4065/81.7.973 16835977

[B255] Yee KW O'Brien SM Chronic lymphocytic leukemia: diagnosis and treatment Mayo Clin Proc 2006 81 8 1105 1129 10.4065/81.8.1105 16901035

[B256] Kay NE Hamblin TJ Jelinek DF Dewald GW Byrd JC Farag S Lucas M Lin T Chronic lymphocytic leukemia Hematology Am Soc Hematol Educ Program 2002 193 213 12446424 10.1182/asheducation-2002.1.193

[B257] Fagioli F Zecca M Locatelli F Lanino E Uderzo C Di Bartolomeo P Berger M Favre C Rondelli R Pession A Allogeneic stem cell transplantation for children with acute myeloid leukemia in second complete remission J Pediatr Hematol Oncol 2008 30 8 575 583 10.1097/MPH.0b013e31816e2342 18799933

[B258] Frassoni F Gualandi F Podesta M Raiola AM Ibatici A Piaggio G Sessarego M Sessarego N Gobbi M Sacchi N Direct intrabone transplant of unrelated cord-blood cells in acute leukaemia: a phase I/II study Lancet Oncol 2008 9 9 831 839 10.1016/S1470-2045(08)70180-3 18693069

[B259] Ruiz-Arguelles GJ Gomez-Almaguer D Morales-Toquero A Gutierrez-Aguirre CH Vela-Ojeda J Garcia-Ruiz-Esparza MA Manzano C Karduss A Sumoza A de-Souza C The early referral for reduced-intensity stem cell transplantation in patients with Ph1 (+) chronic myelogenous leukemia in chronic phase in the imatinib era: results of the Latin American Cooperative Oncohematology Group (LACOHG) prospective, multicenter study Bone Marrow Transplant 2005 36 12 1043 1047 10.1038/sj.bmt.1705190 16247424

[B260] Oehler VG Radich JP Storer B Blume KG Chauncey T Clift R Snyder DS Forman SJ Flowers ME Martin P Randomized trial of allogeneic related bone marrow transplantation versus peripheral blood stem cell transplantation for chronic myeloid leukemia Biol Blood Marrow Transplant 2005 11 2 85 92 10.1016/j.bbmt.2004.09.010 15682068

[B261] Ohnishi K Ino A Kishimoto Y Usui N Shimazaki C Ohtake S Taguchi H Yagasaki F Tomonaga M Hotta T Multicenter prospective study of interferon alpha versus allogeneic stem cell transplantation for patients with new diagnoses of chronic myelogenous leukemia Int J Hematol 2004 79 4 345 353 10.1532/IJH97.03160 15218963

[B262] Das M Saikia TK Advani SH Parikh PM Tawde S Use of a reduced-intensity conditioning regimen for allogeneic transplantation in patients with chronic myeloid leukemia Bone Marrow Transplant 2003 32 2 125 129 10.1038/sj.bmt.1704107 12838275

[B263] Mohty M Labopin M Tabrizzi R Theorin N Fauser AA Rambaldi A Maertens J Slavin S Majolino I Nagler A Reduced intensity conditioning allogeneic stem cell transplantation for adult patients with acute lymphoblastic leukemia: a retrospective study from the European Group for Blood and Marrow Transplantation Haematologica 2008 93 2 303 306 10.3324/haematol.11960 18245655

[B264] Tobinai K Takeyama K Arima F Aikawa K Kobayashi T Hanada S Kasai M Ogura M Sueoka E Mukai K Phase II study of chemotherapy and stem cell transplantation for adult acute lymphoblastic leukemia or lymphoblastic lymphoma: Japan Clinical Oncology Group Study 9004 Cancer Sci 2007 98 9 1350 1357 10.1111/j.1349-7006.2007.00556.x 17640299 PMC11158694

[B265] Isidori A Motta MR Tani M Terragna C Zinzani P Curti A Rizzi S Taioli S Giudice V D'Addio A Positive selection and transplantation of autologous highly purified CD133(+) stem cells in resistant/relapsed chronic lymphocytic leukemia patients results in rapid hematopoietic reconstitution without an adequate leukemic cell purging Biol Blood Marrow Transplant 2007 13 10 1224 1232 10.1016/j.bbmt.2007.07.004 17889360

[B266] Grigg AP Gibson J Bardy PG Reynolds J Shuttleworth P Koelmeyer RL Szer J Roberts AW To LB Kennedy G A prospective multicenter trial of peripheral blood stem cell sibling allografts for acute myeloid leukemia in first complete remission using fludarabine-cyclophosphamide reduced intensity conditioning Biol Blood Marrow Transplant 2007 13 5 560 567 10.1016/j.bbmt.2006.12.449 17448915

[B267] Gutierrez-Aguirre CH Gomez-Almaguer D Cantu-Rodriguez OG Gonzalez-Llano O Jaime-Perez JC Herena-Perez S Manzano CA Estrada-Gomez R Gonzalez-Carrillo ML Ruiz-Arguelles GJ Non-myeloablative stem cell transplantation in patients with relapsed acute lymphoblastic leukemia: results of a multicenter study Bone Marrow Transplant 2007 40 6 535 539 10.1038/sj.bmt.1705769 17618317

[B268] Dreger P Brand R Hansz J Milligan D Corradini P Finke J Deliliers GL Martino R Russell N Van Biezen A Treatment-related mortality and graft-versus-leukemia activity after allogeneic stem cell transplantation for chronic lymphocytic leukemia using intensity-reduced conditioning Leukemia 2003 17 5 841 848 10.1038/sj.leu.2402905 12750695

[B269] Marina Cavazzana-Calvo GC George Q Daley De Luca Michele Ira J Fox Gerstle Claude Robert A Goldstein GH Katherine A High Hyun Ok Kim Hin Peng Lee Ephrat Levy-Lahad Lingsong Li BL Daniel R Marshak Angela McNab Munsie Megan Nakauchi Hiromitsu Mahendra Rao Carlos Simon Valles Srivastava Alok Sugarman Jeremy Patrick L Taylor Veiga Anna Zoloth Laurie Wong AL Research ISfSC Guidelines for the Clinical Translation of Stem Cells 2008 19

[B270] Daley GQ Stem cells: roadmap to the clinic J Clin Invest 120 1 8 10 10.1172/JCI41801 20051631 PMC2798707

[B271] Watt FM Driskell RR The therapeutic potential of stem cells Philos Trans R Soc Lond B Biol Sci 365 1537 155 163 20008393 10.1098/rstb.2009.0149 PMC2842697

[B272] Trounson A New perspectives in human stem cell therapeutic research BMC Med 2009 7 29 10.1186/1741-7015-7-29 19519878 PMC2702289

[B273] Kroon E Martinson LA Kadoya K Bang AG Kelly OG Eliazer S Young H Richardson M Smart NG Cunningham J Pancreatic endoderm derived from human embryonic stem cells generates glucose-responsive insulin-secreting cells in vivo Nat Biotechnol 2008 26 4 443 452 10.1038/nbt1393 18288110

[B274] Preynat-Seauve O Burkhard PR Villard J Zingg W Ginovart N Feki A Dubois-Dauphin M Hurst SA Mauron A Jaconi M Pluripotent stem cells as new drugs? The example of Parkinson's disease Int J Pharm 2009 381 2 113 121 10.1016/j.ijpharm.2009.03.003 19782880

[B275] Burt RK Loh Y Cohen B Stefoski D Balabanov R Katsamakis G Oyama Y Russell EJ Stern J Muraro P Autologous non-myeloablative haemopoietic stem cell transplantation in relapsing-remitting multiple sclerosis: a phase I/II study Lancet Neurol 2009 8 3 244 253 10.1016/S1474-4422(09)70017-1 19186105

[B276] Crop MJ Baan CC Korevaar SS Ijzermans JN Alwayn IP Weimar W Hoogduijn MJ Donor-derived mesenchymal stem cells suppress alloreactivity of kidney transplant patients Transplantation 2009 87 6 896 906 10.1097/TP.0b013e31819b3d72 19300194

[B277] Troeger A Meisel R Moritz T Dilloo D Immunotherapy in allogeneic hematopoietic stem cell transplantation--not just a case for effector cells Bone Marrow Transplant 2005 35 Suppl 1 S59 64 10.1038/sj.bmt.1704849 15812533

[B278] Le Blanc K Frassoni F Ball L Locatelli F Roelofs H Lewis I Lanino E Sundberg B Bernardo ME Remberger M Mesenchymal stem cells for treatment of steroid-resistant, severe, acute graft-versus-host disease: a phase II study Lancet 2008 371 9624 1579 1586 10.1016/S0140-6736(08)60690-X 18468541

[B279] Iyer SS Co C Rojas M Mesenchymal stem cells and inflammatory lung diseases Panminerva Med 2009 51 1 5 16 19352305

[B280] Nasef A Ashammakhi N Fouillard L Immunomodulatory effect of mesenchymal stromal cells: possible mechanisms Regen Med 2008 3 4 531 546 10.2217/17460751.3.4.531 18588475

[B281] Yamanaka S A fresh look at iPS cells Cell 2009 137 1 13 17 10.1016/j.cell.2009.03.034 19345179

[B282] Zhou H Wu S Joo JY Zhu S Han DW Lin T Trauger S Bien G Yao S Zhu Y Generation of induced pluripotent stem cells using recombinant proteins Cell Stem Cell 2009 4 5 381 384 10.1016/j.stem.2009.04.005 19398399 PMC10182564

[B283] Yamashita JK ES and iPS cell research for cardiovascular regeneration Exp Cell Res 2010 316 16 2555 2559 10.1016/j.yexcr.2010.04.004 20385126

[B284] Foster KW Liu Z Nail CD Li X Fitzgerald TJ Bailey SK Frost AR Louro ID Townes TM Paterson AJ Induction of KLF4 in basal keratinocytes blocks the proliferation-differentiation switch and initiates squamous epithelial dysplasia Oncogene 2005 24 9 1491 1500 10.1038/sj.onc.1208307 15674344 PMC1361530

[B285] Hochedlinger K Yamada Y Beard C Jaenisch R Ectopic expression of Oct-4 blocks progenitor-cell differentiation and causes dysplasia in epithelial tissues Cell 2005 121 3 465 477 10.1016/j.cell.2005.02.018 15882627

[B286] Nair V Retrovirus-induced oncogenesis and safety of retroviral vectors Curr Opin Mol Ther 2008 10 5 431 438 18830918

[B287] Soldner F Hockemeyer D Beard C Gao Q Bell GW Cook EG Hargus G Blak A Cooper O Mitalipova M Parkinson's disease patient-derived induced pluripotent stem cells free of viral reprogramming factors Cell 2009 136 5 964 977 10.1016/j.cell.2009.02.013 19269371 PMC2787236

[B288] Maherali N Hochedlinger K Guidelines and techniques for the generation of induced pluripotent stem cells Cell Stem Cell 2008 3 6 595 605 10.1016/j.stem.2008.11.008 19041776

[B289] Tenzen T Zembowicz F Cowan CA Genome modification in human embryonic stem cells J Cell Physiol 2010 222 2 278 281 10.1002/jcp.21948 19877154

[B290] Zarzeczny A Scott C Hyun I Bennett J Chandler J Charge S Heine H Isasi R Kato K Lovell-Badge R iPS cells: mapping the policy issues Cell 2009 139 6 1032 1037 10.1016/j.cell.2009.11.039 20005794

[B291] Lewis R Zhdanov RI Centenarians as stem cell donors Am J Bioeth 2009 9 11 1 3 19882441 10.1080/15265160903320554

